# Alarmin-painted exosomes elicit persistent antitumor immunity in large established tumors in mice

**DOI:** 10.1038/s41467-020-15569-2

**Published:** 2020-04-14

**Authors:** Bingfeng Zuo, Han Qi, Zhen Lu, Lu Chen, Bo Sun, Rong Yang, Yang Zhang, Zhili Liu, Xianjun Gao, Abin You, Li Wu, Renwei Jing, Qibing Zhou, HaiFang Yin

**Affiliations:** 1 Key Laboratory of Immune Microenvironment and Disease (Ministry of Education) & Tianjin Key Laboratory of Cellular Homeostasis and Human Diseases & Department of Cell Biology, Tianjin Medical University, Qixiangtai Road, Heping District, Tianjin, 300070 China; 2 Department of Hepatobiliary, Tianjin Medical University Cancer Institute and Hospital, National Clinical Research Center for Cancer, Key Laboratory of Cancer Prevention and Therapy, Tianjin, 300060 China; 3 Department of The Second Department of Breast Cancer, Tianjin Medical University Cancer Institute and Hospital, National Clinical Research Center for Cancer, Key Laboratory of Cancer Prevention and Therapy, Tianjin, 300060 China; 4 Department of Nanomedicine & Biopharmaceuticals, National Engineering Research Center for Nanomedicine, Huazhong University of Science and Technology, Wuhan, 430074 Hubei Province China

**Keywords:** Cancer therapy, Cancer immunotherapy

## Abstract

Treating large established tumors is challenging for dendritic cell (DC)-based immunotherapy. DC activation with tumor cell-derived exosomes (TEXs) carrying multiple tumor-associated antigen can enhance tumor recognition. Adding a potent adjuvant, high mobility group nucleosome-binding protein 1 (HMGN1), boosts DCs’ ability to activate T cells and improves vaccine efficiency. Here, we demonstrate that TEXs painted with the functional domain of HMGN1 (TEX-N1ND) via an exosomal anchor peptide potentiates DC immunogenicity. TEX-N1ND pulsed DCs (DC_TEX-N1ND_) elicit long-lasting antitumor immunity and tumor suppression in different syngeneic mouse models with large tumor burdens, most notably large, poorly immunogenic orthotopic hepatocellular carcinoma (HCC). DC_TEX-N1ND_ show increased homing to lymphoid tissues and contribute to augmented memory T cells. Importantly, N1ND-painted serum exosomes from cancer patients also promote DC activation. Our study demonstrates the potency of TEX-N1ND to strengthen DC immunogenicity and to suppress large established tumors, and thus provides an avenue to improve DC-based immunotherapy.

## Abstract (web summary)

The use of tumour exosome-activated dendritic cell (DC) immunotherapy shows promise for the treatment of large established tumours. Here, the authors generate alarmin HMGN1-attached tumour exosomes which significantly improve therapy efficacy by boosting DC activation in several preclinical mouse models.

## Introduction

Dendritic cell (DC)-based cancer immunotherapy relies on activation of DCs as antigen-presenting cells by tumor antigens or lysates to stimulate an adaptive immunological response against tumors. This has shown promise for cancer patients with sipuleucel-T, which was approved for advanced prostate cancer^1,2^, as a typical example. However, DC vaccines currently being investigated in trials are somewhat underwhelming with the overall objective response rate rarely exceeding 15%^3^. However, DC vaccines have good safety profiles and the potential to induce innate and adoptive antitumor immunity for long-term efficacy, thus one of the emerging trends in DC vaccine research is to improve the immunogenicity of next-generation DC vaccines^4^ according to registered DC clinical trials. Inducers that enhance the immunogenicity of exogenous DCs through ex vivo activation of these cells prior to immunization are under intensive scrutiny^5^, and the implementation of next-generation DC vaccines with improved activities might hold the key to fulfilling the therapeutic potential of DC-based immunotherapy.

Maturation cocktails and other immunoadjuvants can be used to promote the immunostimulatory activity of exogenously activated DCs^6–8^. The nature of immunoadjuvants determines the type, the magnitude, the breadth, and the quality of antitumor immune response^9^. DC vaccines that induce a bias towards a Th1 immune response appear to elicit stronger activation of tumor-specific cytotoxic T lymphocytes and natural killer (NK) cells and only tumor-associated antigen (TAA)-specific Th1 immune responses are protective against tumors^10,11^, thus adjuvants that promote this bias are preferred. High-mobility group nucleosome-binding protein 1 (HMGN1) improves DC maturation and activation in response to exogenous antigens and consistently induces Th1 immune responses^12^. Hence HMGN1 has been harnessed to promote generation of antitumor immune responses as DNA vaccines or in combination with chemotherapeutic agents and activators/checkpoint inhibitors in different tumor models^13–15^. However, only limited effects were achieved with DNA vaccines and combinatorial therapy since HMGN1 required intratumoral administration, which is not clinically applicable to most tumors. Therefore, efficient systemic delivery of HMGN1 to cells is needed to unlock the full therapeutic potential of HMGN1 as an immunoadjuvant.

Recently, tumor-derived exosomes (TEXs), which harbor multiple TAAs to provide a broad spectrum of antigen presentation, have drawn much interest as antigens for DC-based immunotherapy^16–18^. TEXs, particularly patient-derived TEXs, have good biocompatibility, low immunogenicity, and high penetration of biological barriers, thus TEXs are good activators of DC vaccines. It was previously shown that covalent linkage of an alarmin to a target TAA could better promote TAA-specific immune responses and immunoprotection^13,14^. Ideally, a method that combines delivery of molecular adjuvants with the TAA-enriched exosomes would improve the immunogenicity of DC immunotherapy and consequently, potential therapeutic outcome.

Here, we covalently link the functional N-terminal domain of HMGN1 (N1ND) to CP05, a recently identified exosomal anchor peptide which allows direct and efficient cargo loading on exosomes^12,19^, and paint N1ND on TEXs (TEX-N1ND). DCs pulsed with TEX-N1ND (DC_TEX-N1ND_) elicit durable tumor retardation in multiple syngeneic subcutaneous tumor models bearing large, established tumor burdens, and also in poorly immunogenic orthotopic hepatocellular carcinoma (HCC) mice with large tumor loads. Importantly, TEX-N1ND also augments human DC immunogenicity against different patient-derived cancer cells in vitro. Altogether TEX-N1ND heightens the immunostimulatory activities of DCs and migration to lymph nodes, consequently accelerating the generation of long-lived protective T cell memory response, which is responsible for the persistent antitumor immunity of activated DCs.

## Results

### N1ND-painted TEX promotes N1ND uptake and DCs’ immunity

**Fig. 1:**
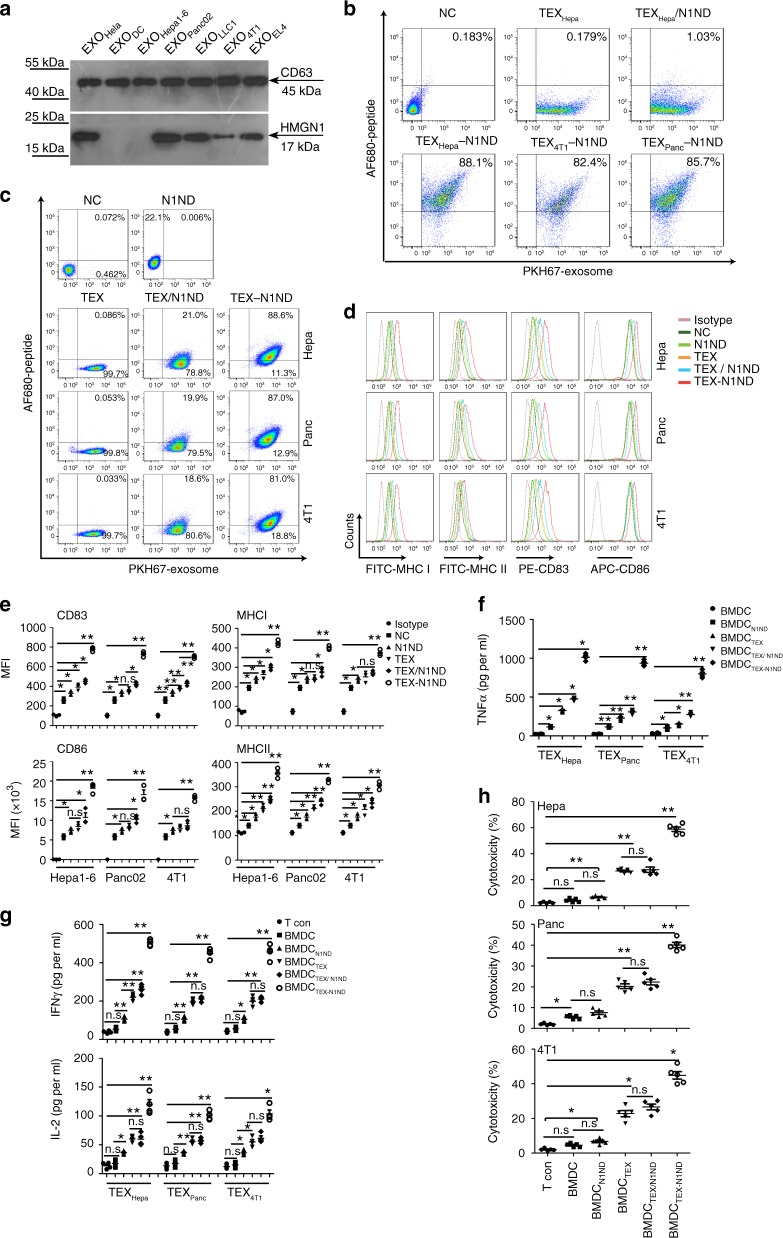
HMGN1 expression in TEXs and in vitro assessment of TEX-N1ND. **a** Western blot analysis for detecting the expression of HMGN1 in TEXs derived from different tumor cells. Total protein (20 μg) was loaded for TEXs. Exosomes from Hela (EXO_Hela_) and DCs (EXO_DC_) were used as positive and negative controls, respectively. CD63 was used as an exosomal marker protein. This experiment was repeated once (two in total). **b** Quantitative analysis on the binding efficiency of N1ND-CP05 to TEXs. TEX_Hepa_/N1ND or TEX_Hepa_-N1ND represents the mixture of N1ND and TEXs from Hepa1-6 cells (TEX_Hepa_) or the complex of N1ND-CP05 with TEX_Hepa_, respectively. AF680-labeled N1ND-CP05 or N1ND peptide (80 μg) was incubated with PKH67-labeled TEXs (40 μg) for 12 h. Free peptide was removed by diafiltration. NC refers to unlabeled TEX_Hepa_. **c** Flow cytometric analysis of TEX-N1ND uptake in BMDCs at 24 h after incubation. NC refers to untreated DCs. Levels (**d**) and quantitative analysis (**e**) of surface markers and co-stimulatory molecules in BMDC_TEX-N1ND_, BMDC_TEX/N1ND_, or BMDC_TEX_ (*n* = 3; one-way ANOVA post hoc Student–Newman–Keuls test). NC refers to untreated DCs. **f** Measurement of TNF-α secretion in supernatants of BMDC_TEX_, BMDC_TEX/N1ND_, or BMDC_TEX-N1ND_ (*n* = 4; one-way ANOVA on ranks). **g** Analysis of IFN-γ and IL-2 in supernatants from splenic T lymphocytes activated by BMDC_TEX-N1ND_, BMDC_TEX/N1ND_, or BMDC_TEX_ (*n* = 4; one-way ANOVA post hoc Student–Newman–Keuls test). **h** Cytolysis rate against different murine tumor cells with T cells activated by BMDC_TEX-N1ND_, BMDC_TEX/N1ND_ or BMDC_TEX_, respectively. A LDH-releasing cytotoxic assay was performed to measure the cytolysis efficiency of effector T cells activated by BMDC_TEX-N1ND_, BMDC_TEX/N1ND_, or BMDC_TEX_, respectively in Hepa1-6, Panc02 (*n* = 5; one-way ANOVA post hoc Student–Newman–Keuls test) and 4T1 cells (one-way ANOVA on ranks). *N* refers to the number of individual biological replicate unless otherwise specified. Data are presented as means ± s.e.m. (*p < 0.05; **p < 0.001; n.s not significant). Source data are provided as a Source Data file.

Motivated by HMGN1’s efficacy at inducing Th1 antitumor immune response and our recently discovered strategy for intracellular delivery of cargoes^12,13,19^, we explored the feasibility of painting the surface of TEX with the functional N-terminus of HMGN1 (N1ND) using CD63-binding peptide (CP05) (TEX-N1ND). Incubation of DCs with TEX-N1ND can deliver N1ND into cells to enhance TEX-mediated DC activation and immunogenicity in different tumor models. To determine whether preexisting HMGN1 levels in TEXs would have any impact on the effect of additional N1ND on DC activation, we assayed TEXs, with a buoyant density of 1.13–1.23 g ml^−1^ (Supplementary Fig. 1a), from murine pancreatic cancer (Panc02), Lewis lung cancer (LLC1), lymphoma (EL4), breast cancer (4T1), HCC (Hepa1-6) cells, and DCs for HMGN1 (Fig. 1a). The levels of HMGN1 in TEXs generally correlated with their parental cells (Supplementary Fig. 1b). TEXs from Hepa1-6, 4T1, and Panc02 were selected as representative TEXs containing no, medium, or high levels of HMGN1 respectively for subsequent studies. AF680-labeled N1ND-CP05 efficiently bound to TEXs, with the highest binding efficiency to TEX_Hepa_ (88.1%) (Fig. 1b), correlating with the percentage of CD63-positive TEXs (Supplementary Fig. 1c) without altering the morphology of TEXs (Supplementary Fig. 1d). In contrast, only trace binding was detected in the mixture of TEX_Hepa_ with N1ND without CP05 (Fig. 1b, TEX_Hepa_/N1ND), demonstrating that N1ND binds TEXs due to CP05. Dramatically increased uptake of AF680-labeled N1ND-CP05 was observed in bone marrow-derived DCs (BMDC) after incubation with TEX (TEX-N1ND) (40 μg ml^−1^) (BMDC_TEX-N1ND_) when compared with the mixture of TEXs with N1ND without CP05 (BMDC_TEX/ N1ND_) or N1ND-CP05 alone (BMDC_N1ND_) (Fig. 1c and Supplementary Fig. 1e), suggesting that binding to TEXs enabled efficient delivery of N1ND-CP05 to cells. Major histocompatibility complex class (MHC) I/II, co-stimulatory molecules (Fig. 1d, e) and tumor necrosis factor-α (TNF-α) (Fig. 1f), a proinflammatory cytokine induced by HMGN1, were significantly upregulated in DCs treated with TEX-N1ND compared with the controls. Importantly, TEX-N1ND induced comparable DC activation in different TEXs independent of endogenous HMGN1 levels. Secreted levels of interferon-γ (IFN-γ) and interleukin-2 (IL-2) consistently increased in splenic naive T cells stimulated with BMDC_TEX-N1ND_ (Fig. 1g), resulting in significantly higher cytolysis rates against parental tumor cells, from which TEXs were derived, compared with other controls (Fig. 1h), indicating that N1ND is capable of activating DCs in vitro and increasing the immunogenicity of the DCs.

### N1ND enhances TEX-induced DC immunity on large tumors

**Fig. 2:**
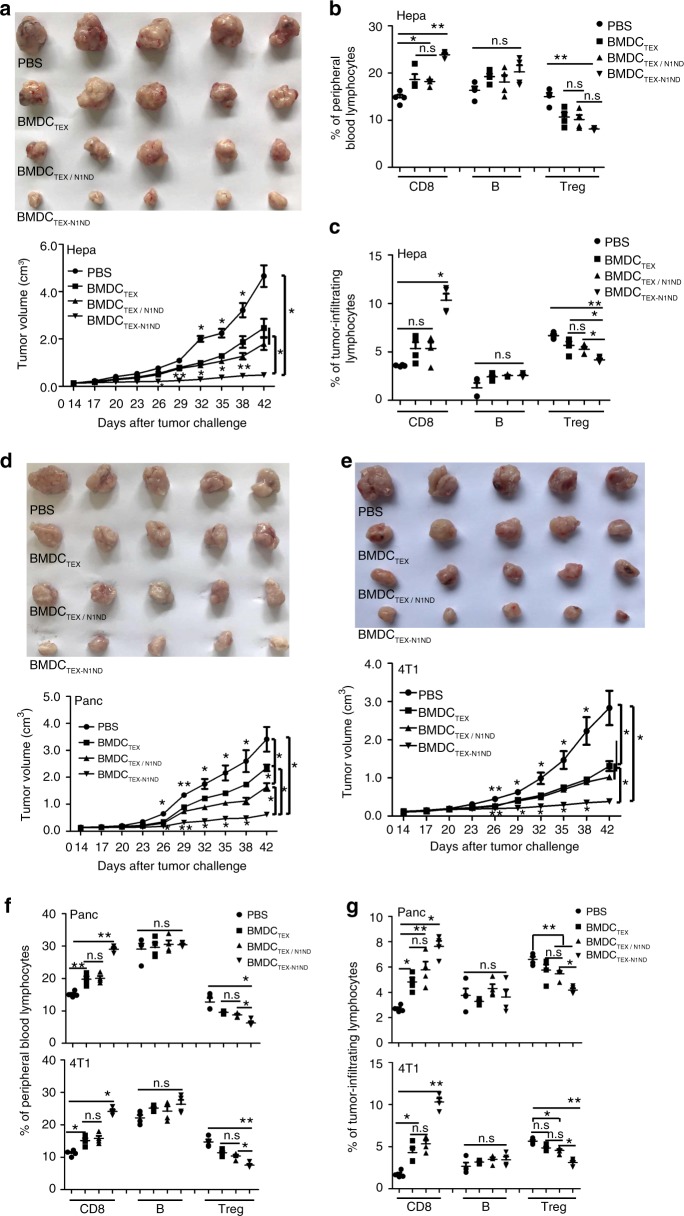
Effect of BMDC_TEX-N1ND_ on large subcutaneous tumors. **a** Measurement of tumor volume in subcutaneous *C57BL/6* HCC mice treated with BMDC_TEX-N1ND_, BMDC_TEX/N1ND_, or BMDC_TEX_ (2 × 10^6^ cells once per week for 3 weeks) at day 26 (*n* = 5; one-way ANOVA on ranks), day 29 (*n* = 5; one-way ANOVA post hoc Student–Newman–Keuls test), day 32 and day 35 (*n* = 5; one-way ANOVA on ranks), day 38 (*n* = 5; one-way ANOVA post hoc Student–Newman–Keuls test) and day 42 (*n* = 5; one-way ANOVA on ranks). TEXs were derived from murine Hepa1-6 cells. Flow cytometric analysis of different immune cells in blood (**b**) or tumor tissues (**c**) from HCC mice treated with BMDC_TEX-N1ND_, BMDC_TEX/N1ND_, or BMDC_TEX_, respectively (*n* = 4; one-way ANOVA post hoc Student–Newman–Keuls test; *n* represents the number of animals used for each group). Measurement of tumor volume in syngeneic subcutaneous *C57BL/6* pancreatic cancer mice (**d**) or *BALB/C* breast cancer mice (**e**) treated with BMDC_TEX-N1ND_, BMDC_TEX/N1ND_, or BMDC_TEX_ (2 × 10^6^ cells once per week for 3 weeks) day 26 (*n* = 5; one-way ANOVA post hoc Student–Newman–Keuls test), day 29 (*n* = 5; one-way ANOVA post hoc Student–Newman–Keuls test), day 32, 35, and 42 (*n* = 5; one-way ANOVA on ranks). TEXs were derived from murine pancreatic or breast cancer cells. Flow cytometric analysis of different immune cells in blood (**f**) or tumor tissues (**g**) from subcutaneous tumor mice treated with BMDC_TEX-N1ND_, BMDC_TEX/N1ND_, or BMDC_TEX_ (*n* = 4; one-way ANOVA post hoc Student–Newman–Keuls test). Data are presented as means ± s.e.m. (*p < 0.05; **p < 0.001; n.s not significant). Source data are provided as a Source Data file.

Treating large established tumors, which closely mimic the typical clinical presentation of patients, remains a challenge for initial DC-based immunotherapy, thus we evaluated BMDC_TEX-N1ND_ in three different subcutaneous syngeneic tumor models: Hepa1-6 HCC, Panc02 pancreatic cancer, and 4T1 breast cancer. Tumor cells (2 × 10^6^) were injected subcutaneously and allowed to grow for 14 days to ~40–60 mm^2^ depending on tumor type before initiation of therapy. BMDC_TEX-N1ND_, BMDC_TEX/N1ND_, or BMDC_TEX_ (2 × 10^6^) were administered intravenously once per week for 3 weeks, a protocol identical to our previous study^18^. Robust and persistent tumor growth inhibition with very similar residual and significantly smaller tumor sizes than other groups (Fig. 2a) was achieved in HCC tumor-bearing mice treated with BMDC_TEX-N1ND_ 42 days after tumor challenge; whereas BMDC_TEX/N1ND_ or BMDC_TEX_ elicited weaker responses with higher variability. Increased CD8^+^ and reduced CD4^+^CD25^+^ regulatory T cells (Tregs) were consistently detected in blood and tumor tissues from mice treated with BMDC_TEX-N1ND_ compared with other groups (Fig. 2b, c and Supplementary Fig. 2a, b). In contrast, no significant change was found for B cells across different treatment groups (Fig. 2b, c). Similarly, durable tumor retardation was observed for Panc02 and 4T1 subcutaneous tumor-bearing mice treated with BMDC_TEX-N1ND_ compared with other groups on day 42 (Fig. 2d, e) with similar shifts in circulatory and tumor CD8^+^ T cells and Tregs (Fig. 2f, g), suggesting that the strategy is broadly applicable across tumor types. Notably, consistent with the previous study^18^, a cross-protective effect was observed in mice bearing subcutaneous HCC treated with DC_TEX(Panc)-N1ND_ (Supplementary Fig. 2c, d), and the presence of N1ND did not influence the antigen-specific immune response elicited by TEXs as demonstrated by significant increases in alpha-fetoprotein (AFP)-specific CD8^+^ T cells (Supplementary Fig. 2e) and in levels of IFN-γ and IL-2 upon stimulation with AFP212 (Supplementary Fig. 2f), an identified murine AFP epitope^20^, in culture supernatants of splenic T lymphocytes from mice bearing subcutaneous HCC treated with DC_TEX(Hepa)-N1ND_ but not with DC_TEX(Panc)_- or DC_TEX(4T1)-N1ND_. Importantly, a hierarchy of efficacy was observed with different BMDC_TEX-N1ND_ in three tumor models and was inversely correlated with the endogenous expression of HMGN1 in TEXs, with TEXs derived from HCC cells showing the greatest efficacy, implying that DC_TEX-N1ND_ is likely more effective for tumors without or with lower expression of HMGN1.

### DC_TEX-N1ND_ induce persistent retardation of orthotopic HCC

**Fig. 3:**
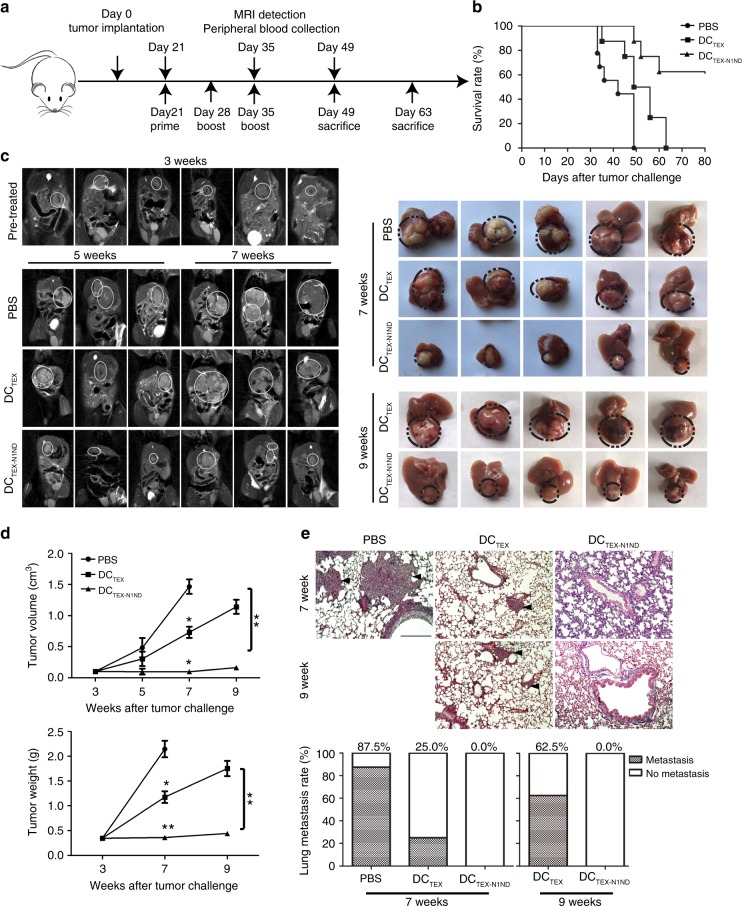
Antitumor effect of DC_TEX-N1ND_ in orthotopic HCC mice (H-2^b^). Day-21 orthotopic *C57BL6* HCC mice were intravenously treated with PBS(black circles), DC_TEX_ (black squares), or DC_TEX-N1ND_ (black triangles) (2 × 10^6^ cells once per week for 3 weeks). **a** Schematic diagram for the dosing regimen of DC_TEX-N1ND_ in day-21 orthotopic *C57BL/6* HCC mice therapeutically. **b** Survival rate of day-21 orthotopic HCC mice treated with PBS (*n* = 9), DC_TEX_ (*n* = 8), or DC_TEX-N1ND_ (*n* = 8), respectively. **c** Real-time MRI monitoring and measurement of tumor nodules in day-21 orthotopic HCC mice treated with PBS, DC_TEX_ or DC_TEX-N1ND_ at different time-points. **d** Analysis of tumor volume and weight from orthotopic HCC mice treated with PBS, DC_TEX_, or DC_TEX-N1ND_ on week 5 (*n* = 3), week 7 (*n* = 11 for PBS; *n* = 12 for DC_TEX_ and DC_TEX-N1ND_; one-way ANOVA on ranks), and 9 (*n* = 11 for DC_TEX_ and *n* = 12 for DC_TEX-N1ND_; two-tailed Mann–Whitney *U* test) (for pretreated controls on week 3, *n* = 7). The experiments for week 7 and 9 were repeated once (two in total). **e** Histological examination and quantitative analysis of pulmonary metastasis of HCC in lungs from orthotopic HCC mice treated with PBS, DC_TEX_, or DC_TEX-N1ND_ on week 7 and 9, respectively (scale bar = 200 μm) (*n* = 8). Arrowheads point to HCC nodules. Data are presented as means ± s.e.m. (*p < 0.05; **p < 0.001). Source data are provided as a Source Data file.

To understand the cellular and molecular mechanisms underlying the observed therapeutic effect of DC_TEX-N1ND_, we analyzed responses to DC_TEX-N1ND_ in an immunosuppressive, poorly immunogenic Hepa1-6 HCC model. Subcutaneous tumors do not faithfully recapitulate the histology and complexity of HCC, thus we employed two different orthotopic HCC mouse models bearing large tumor loads. A day-7 HCC model was generated by transplanting more tumor tissues to accelerate the formation of large tumors (0.68 ± 0.02 in longitudinal diameter) within 7 days, and another with tumor loads (0.55 ± 0.04 in longitudinal diameter) formed in 21 days (day-21) by transplanting fewer tumor tissues to allow the formation of immunosuppressive microenvironment (Supplementary Fig. 3a). Tumors grew progressively in day-7 orthotopic HCC mice resulting in 100% lethality in untreated mice by day 35, replacing most of the livers (Supplementary Fig. 3b–e). Three weekly intravenous doses of DC_TEX-N1ND_ (2 × 10^6^) significantly inhibited tumor growth and extended the lifespan of tumor-bearing mice as tumor volume and mass were significantly reduced and survival rate increased compared with other groups (Supplementary Fig. 3c–e). Persistent tumor retardation was observed in mice treated with DC_TEX-N1ND_ compared with other groups on day 32 and 46 (Supplementary Fig. 3c, d), demonstrating that N1ND-CP05 enhanced activated DCs’ antitumor immunity. Concordantly, in the day-21 orthotopic HCC mice, DC_TEX-N1ND_ (2 × 10^6^) promoted robust and sustained inhibition of induced tumors compared with mice treated with DC_TEX_, with a 60% survival rate at week 9 vs. 0% for DC_TEX_ (Fig. 3a–d). Real-time monitoring of tumor growth via magnetic resonance imaging (MRI) revealed progressive tumor growth and multiple nodular foci formation in DC_TEX_-treated and untreated mice, whereas no evident tumor progression was found in mice treated with DC_TEX-N1ND_ (Fig. 3c, d), demonstrating that DC_TEX-N1ND_ results in sustainable tumor inhibition. Strikingly, no pulmonary metastasis was found in tumor-bearing mice treated with DC_TEX-N1ND_ at week 9; in contrast, prominent pulmonary metastasis was established in DC_TEX_-treated mice (Fig. 3e). These findings demonstrate that TEX-N1ND activates DCs to induce greater antitumor immunity.

### DC_TEX-N1ND_ reshape orthotopic HCC microenvironment in mice

**Fig. 4:**
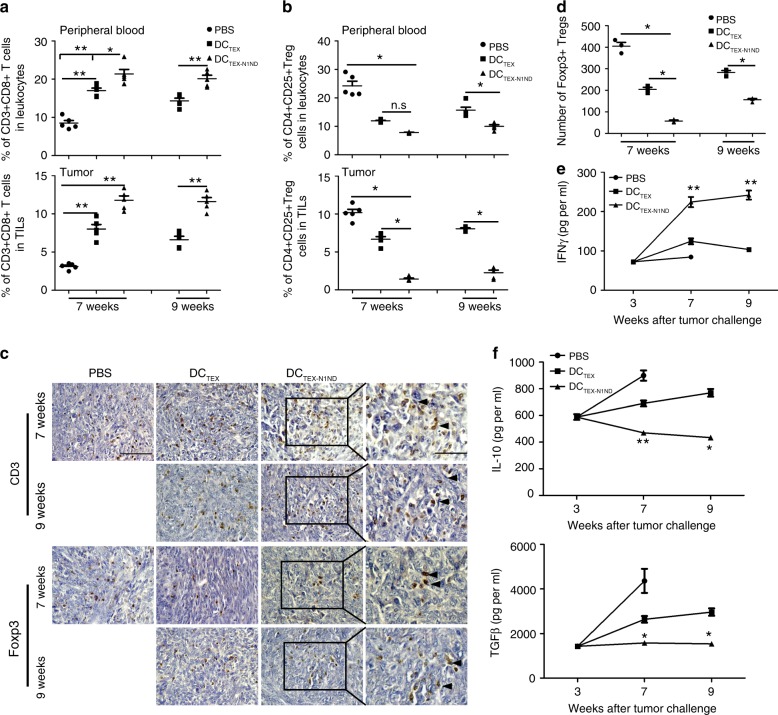
DC_TEX-N1ND_ reshaped orthotopic microenvironment in mice. **a** Flow cytometric analysis of CD8^+^ T lymphocytes in blood and tumor tissues from orthotopic *C57BL/6* HCC mice treated with PBS, DC_TEX_, or DC_TEX-N1ND_ on week 7 (one-way ANOVA post hoc Student–Newman–Keuls test) and 9 (two-tailed *t* test) (*n* = 5). **b** Flow cytometric analysis of CD4^+^CD25^+^ Tregs in blood and tumor tissues from orthotopic *C57BL/6* HCC mice treated with PBS, DC_TEX_, or DC_TEX-N1ND_ on week 7 (one-way ANOVA on ranks) and 9 (two-tailed *t* test) (*n* = 5). **c** Immunohistochemistry of CD3^+^ T cells and Foxp3^+^ Tregs in tumor sections from different treatment samples to determine the extent of T cell infiltration (scale bar = 100 or 50 μm in magnified images). Arrowheads point to CD3^+^ or Foxp3^+^ T cells. This experiment was repeated once (two in total). **d** Quantification of Foxp3^+^ T cells in tumor sections from different treatment groups on week 7 (one-way ANOVA on ranks) and 9 (two-tailed Mann–Whitney *U* test) (*n* = 3; *n* represents the number of animals used for each group). **e** Measurement of IFN-γ in tumor tissues from treated mice with ELISA on week 3 (*n* = 3), week 7 (*n* = 5; one-way ANOVA post hoc Student–Newman–Keuls test), and 9 (*n* = 5; two-tailed *t* test). **f** Measurement of immunosuppressive cytokines including TGF-β on week 3 (*n* = 3), week 7 (one-way ANOVA on ranks), and 9 (two-tailed Mann-Whitney *U* test) (*n* = 5) and IL-10 on week 3 (*n* = 3), 7 (*n* = 5; one-way ANOVA post hoc Student–Newman–Keuls test), and 9 (*n* = 5; two-tailed Mann-Whitney *U* test). *N* represents the number of animals used for each group. The comparison was conducted between DC_TEX-N1ND_ and DC_TEX_ or PBS groups at the same time-point. Data are presented as means ± s.e.m. (*p < 0.05; **p < 0.001; n.s not significant). Source data are provided as a Source Data file.

The tumor microenvironment plays a crucial role in tumor progression and prognosis^21^, therefore modulation of the tumor microenvironment may augment the antitumor immune response. To determine the effect of DC_TEX-N1ND_ on the tumor microenvironment, we examined CD8^+^ T cells in blood and tumor tissues from day-21 orthotopic HCC mice treated with DC_TEX-N1ND_ as DC_TEX_ and HMGN1 were shown to function primarily via stimulation of CD8^+^ T cells and increased IFN-γ secretion^13^. Significant increases in CD8^+^ T cells and declines in CD4^+^CD25^+^ Tregs were detected in blood and tumors from mice treated with DC_TEX-N1ND_ compared with other groups (Fig. 4a, b), indicating that DC_TEX-N1ND_ primarily promotes CD8^+^ T cell priming. Active recruitment of effector CD3^+^ T lymphocytes to primary tumor sites in orthotopic HCC mice was observed in tumor cross-sections from DC_TEX-N1ND_-treated mice compared with DC_TEX_ at different time-points, and fewer effector T cells were detected in PBS-treated mice on week 7 (Fig. 4c). Sporadic and significantly fewer FoxP3^+^ Tregs were found in DC_TEX-N1ND_-treated samples compared with other groups (Fig. 4c, d). Level of IFN-γ significantly rose and TGF-β and IL-10 levels were significantly reduced in tumor tissues and blood from DC_TEX-N1ND_-treated mice, compared with DC_TEX_ and PBS groups (Fig. 4e, f, and Supplementary Fig. 4a, b). Overall, these data demonstrate that DC_TEX-N1ND_ can induce persistent antitumor immunity and reshape tumor microenvironment in orthotopic HCC mice bearing large established tumor burdens.

### TEX-N1ND boosts memory T cells for long-lasting immunity

**Fig. 5:**
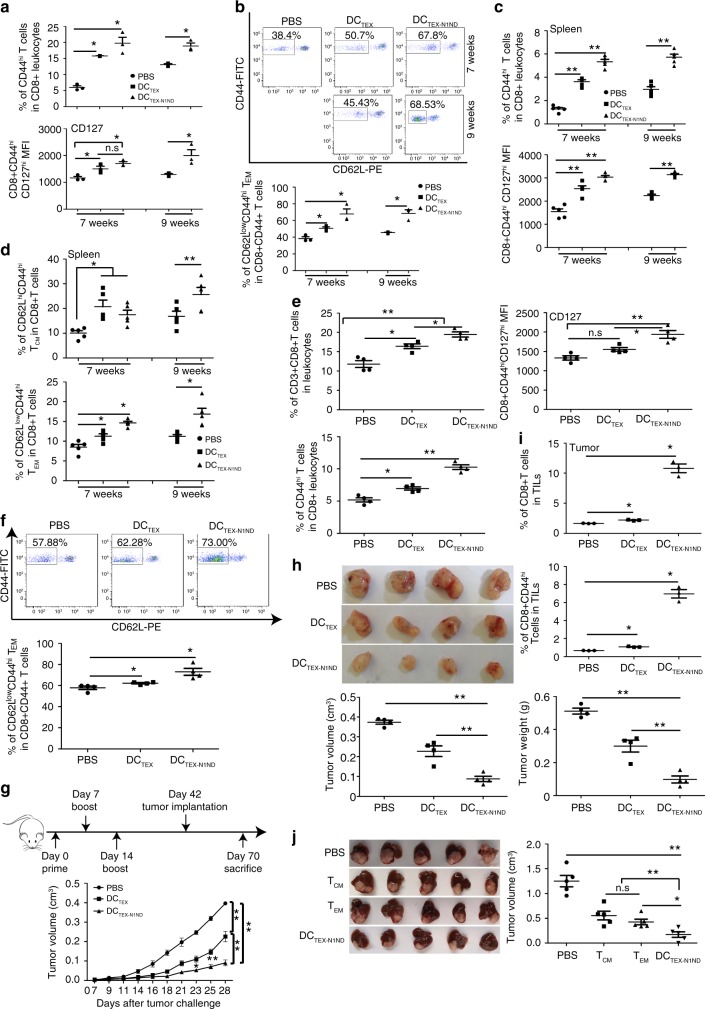
DC_TEX-N1ND_ augmented memory T cells in orthotopic HCC mice. Flow cytometric analysis of long-lived memory T cells (**a**) or T_EM_ cells (**b**) in blood from day-21 orthotopic HCC mice treated with DC_TEX-N1ND_, DC_TEX_ or PBS on week 7 (one-way ANOVA on ranks) and 9 (two-tailed *t* test). CD44^hi^ or CD127^hi^ means CD44^high^ or CD127^high^, respectively (*n* = 3). MFI mean fluorescence intensity. Quantitative analysis of long-lived memory T cells (**c**) or T_EM_ and T_CM_ cells (**d**) in the spleen from day-21 orthotopic HCC mice treated with DC_TEX-N1ND_, DC_TEX_, or PBS on week 7 (one-way ANOVA post hoc Student–Newman–Keuls test) and 9 (two-tailed *t* test), respectively (*n* = 5). CD62L^hi^ represents CD62L^high^. Flow cytometric analysis of effector and memory T cells (**e**) or T_EM_ cells (**f**) in blood from immunized wild-type *C57BL/6* mice before tumor challenge (one-way ANOVA post hoc Student–Newman–Keuls test). These mice were immunized intravenously with DC_TEX-N1ND_, DC_TEX_ (2 × 10^6^ cells once per week for 3 weeks) or PBS, respectively and blood was collected 4 weeks after last immunization before challenge (*n* = 4). Measurement of tumor growth (**g**) or tumor volume/weight (**h**) of immunized *C57BL/6* mice at 4 weeks after challenge (*n* = 4; one-way ANOVA post hoc Student–Newman–Keuls test). **i** Flow cytometric analysis of effector and memory T cells in tumor tissues from *C57BL/6* mice immunized with DC_TEX_ or DC_TEX-N1ND_ 4 weeks after tumor challenge (*n* = 3; one-way ANOVA on ranks). TILs refer to tumor-infiltrating lymphocytes. **j** Measurement of tumor growth of day-7 established orthotopic HCC mice treated with memory T cells isolated from DC_TEX-N1ND_-immunized *C57BL/6* mice (*n* = 5; one-way ANOVA post hoc Student–Newman–Keuls test). DC_TEX-N1ND_ was used as a positive control. Data are presented as means ± s.e.m. (*p < 0.05; **p < 0.001; n.s not significant). Source data are provided as a Source Data file.

Generation of memory CD8^+^ T cells can greatly enhance protective immunity^22^ and thus is an important goal of vaccination. Given the critical role of CD8^+^ T cells in the therapeutic effect, we first analyzed the effect of DC_TEX-N1ND_ on circulatory memory T cells from treated mice. Strikingly, significantly greater amplification of effector and memory (CD8^+^CD44^high^) T cells, particularly CD8^+^CD44^high^CD127^high^ long-lived memory T cells^23–25^, occurred in mice receiving DC_TEX-N1ND_ treatment on week 7 and 9, compared with corresponding DC_TEX_ and PBS treatment groups (Fig. 5a). DC_TEX-N1ND_ treatment also elicited significantly more circulatory CD8^+^ effector memory T cells (T_EM_; CD44^high^CD62L^low^) on week 7 and 9^24^, compared with DC_TEX_ and PBS treatment (Fig. 5b), suggesting that increased T_EM_ cells contributed to the long-lasting protective immunity. Consistently, the proportion of CD8^+^CD44^high^ and CD8^+^CD44^high^CD127^high^ memory T cells, particularly T_EM_ and CD8^+^ central memory T cells (T_CM_; CD44^high^CD62L^high^), significantly increased in the secondary lymphoid tissue (spleen) from DC_TEX-N1ND_- treated mice^24^ compared with other groups (Fig. 5c, d and Supplementary Fig. 5a). To ascertain if memory T cells induced by repeated DC_TEX-N1ND_ vaccination accounted for the prolonged antitumor immunity observed, we immunized *C57BL/6* mice with DC_TEX-N1ND_ (2 × 10^6^) intravenously once per week for 3 weeks. As expected, circulatory effector and memory T cells, particularly long-lived memory T cells, significantly increased in DC_TEX-N1ND_-treated mice, whereas to a lesser extent in DC_TEX_ compared with PBS controls (Fig. 5e and Supplementary Fig. 5b), indicating that DC_TEX-N1ND_ is potent at triggering the generation of memory T cells. Circulatory T_EM_ cells were also significantly elevated in DC_TEX-N1ND_-treated mice, compared with other groups (Fig. 5f). Correspondingly, persistent tumor inhibition and effector T cells infiltration into tumor sites were observed in DC_TEX-N1ND_-immunized *C57BL/6* mice 4 weeks after tumor challenge with Hepa1-6 cells (5 × 10^5^) injected subcutaneously as tumor volume and weight significantly decreased (Fig. 5g, h) and CD8^+^ effector T and T_EM_ cells significantly increased in tumor tissues (Fig. 5i), and memory T cells in blood (Supplementary Fig. 5c, d) and the spleen (Supplementary Fig. 5e) significantly rose. To further confirm the direct involvement of memory T cells in the long-lasting antitumor immunity triggered by DC_TEX-N1ND_, we isolated T_EM_ and T_CM_ from *C57BL/6* mice immunized with DC_TEX-N1ND_ under identical conditions as described above and intravenously administered T_EM_ or T_CM_ (5 × 10^6^) into day-7 orthotopic HCC mice for single injection. Strikingly, tumor growth was significantly inhibited in T_EM_- and T_CM_-treated HCC mice compared with untreated controls (Fig. 5j), strengthening the notion that memory T cell induction mediated protective immunity against the tumor. These findings support the conclusion that memory T cells boosted by DC_TEX-N1ND_ contribute to long-lasting protective immune response.

### DC activation and lymph node homing boosts immune memory

**Fig. 6:**
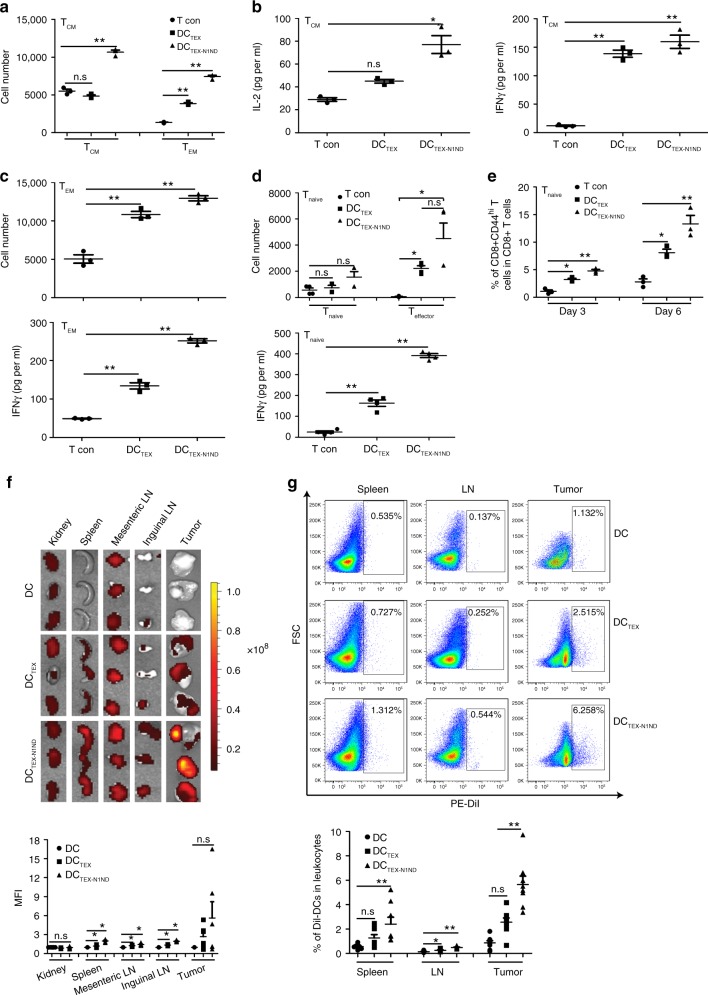
Effect of DC_TEX-N1ND_ on T cells from DC_TEX-N1ND_-immunized mice. *C57BL/6* mice were immunized with DC_TEX-N1ND_ (2 × 10^6^ cells once per week for 3 weeks) and splenic memory or naive T cells were isolated from immunized mice 4 weeks after last immunization. **a** Quantification of T_CM_ and T_EM_ cells in T_CM_ cell populations after the co-incubation of T_CM_ cells with DC_TEX-N1ND_ or DC_TEX_ (3 × 10^4^) for 3 days (*n* = 3; one-way ANOVA post hoc Student–Newman–Keuls test; *n* represents the number of animals immunized with DC_TEX-N1ND_). **b** Levels of IFN-γ and IL-2 in the culture medium after the co-incubation of T_CM_ cells with DC_TEX-N1ND_ or DC_TEX_ (3 × 10^4^) for 3 days (*n* = 3; one-way ANOVA post hoc Student–Newman–Keuls test; *n* represents the number of animals immunized with DC_TEX-N1ND_). **c** Analysis of T_EM_ cells and IFN-γ in T_EM_ cell populations stimulated with DC_TEX-N1ND_ or DC_TEX_ (3 × 10^4^), respectively (*n* = 3; one-way ANOVA post hoc Student–Newman–Keuls test; *n* represents the number of animals immunized with DC_TEX-N1ND_). **d** Analysis of effector T cells (one-way ANOVA on ranks) and IFN-γ (one-way ANOVA post hoc Student–Newman–Keuls test) in naive T cell populations stimulated with DC_TEX-N1ND_ or DC_TEX_ (3 × 10^4^) for 3 or 6 days, respectively (*n* = 4; *n* represents the number of animals used for isolation of naive T cells). **e** Quantification of memory T cells in naive T cell populations after the co-incubation of naive T cells with DC_TEX-N1ND_ or DC_TEX_ (3 × 10^4^) on day 3 and 6, respectively (*n* = 3; one-way ANOVA post hoc Student–Newman–Keuls test; *n* represents the number of animals used for isolation of naive T cells). **f** Tissue distribution and quantification of DiI-labeled DC_TEX-N1ND_ or DC_TEX_ (5 × 10^6^) in orthotopic HCC mice 48 h after single intravenous injection (*n* = 6; one-way ANOVA on ranks). The ratio is relative to untreated DCs. LN lymph node. **g** Flow cytometric analysis of DiI-labeled DC_TEX-N1ND_ or DC_TEX_ in orthotopic HCC mice 48 h after single intravenous injection (*n* = 8; one-way ANOVA post hoc Student–Newman–Keuls test). These experiments were repeated once (two in total). Data are presented as means ± s.e.m. (*p < 0.05; **p < 0.001; n.s not significant). Source data are provided as a Source Data file.

To determine if DC_TEX-N1ND_ has a direct impact on memory T cells, we immunized *C57BL/6* mice with DC_TEX_ (2 × 10^6^) weekly for 3 weeks and harvested splenic T_EM_ and T_CM_ cells 4 weeks after the last immunization and stimulated 3 × 10^5^ of each with DC_TEX-N1ND_ or DC_TEX_ (3 × 10^4^) for 72 h. Stimulation of purified T_CM_ by DC_TEX-N1ND_ resulted in a dramatic increase in T_CM_ and T_EM_ numbers (Fig. 6a) and secreted IL-2 and IFN-γ (Fig. 6b) compared with DC_TEX_ treatment of controls, suggesting that DC_TEX-N1ND_ enables efficient T_CM_ proliferation and differentiation to T_EM_ in response to stimulation. In the purified T_EM_ culture, T_EM_ numbers and IFN-γ significantly rose after DC_TEX-N1ND_ treatment compared with other controls (Fig. 6c), indicating N1ND also triggered robust T_EM_ cell proliferation. To assess the direct impact of DC_TEX-N1ND_ on naive T cells, we incubated DC_TEX-N1ND_ or DC_TEX_ with naive T cells for 3 or 6 days. Effector T cells and IFN-γ were significantly elevated in DC_TEX-N1ND_- and DC_TEX_-treated naive T cells, though to a lesser extent with DC_TEX_, compared with untreated T cell controls (Fig. 6d). Examination of memory T cells on day 3 and 6 revealed significantly increased memory T cells in DC_TEX-N1ND_, compared with the controls (Fig. 6e), indicating that DC_TEX-N1ND_ promotes accelerated generation of effector T cells early on which then promotes the differentiation of memory T cells. These findings demonstrate that DC_TEX-N1ND_ augments generation of effector and memory T cells and results in amplification of immunological memory.

Migration of DC to regional lymph nodes, a function of DC activation, is a critical factor influencing therapeutic outcomes of DC vaccines^26,27^. To examine TEX-N1ND’s effect on exogenous DC migration to lymph nodes, we tracked the tissue distribution of DiI-labeled DCs (5 × 10^6^) in tumor-bearing mice 48 h after single intravenous injection. IVIS analysis (Fig. 6f) and flow cytometry of extracted tissues (Fig. 6g and Supplementary Fig. 6) indicated more DCs migrated to the tumor and secondary lymphoid tissues including spleen, mesenteric, and inguinal lymph nodes in DC_TEX-N1ND_-treated mice compared with DC_TEX_ and controls. These findings strengthen the conclusion that TEX-N1ND endows DCs with stronger immunogenicity and homing capacity to lymph nodes and results in boosted generation of effector and memory T cells, which accounts for the long-lasting antitumor immunity.

Since HMGN1 was known to function through toll-like receptor 4 (TLR4)^12^, TLR4-dependent signaling pathway was examined. Expression of TLR4, tumor necrosis factor receptor-associated factor 6 (TRAF6) and myeloid differentiation primary response gene 88 (MYD88) were upregulated in DC_TEX_ and further enhanced by the presence of N1ND (DC_TEX-N1ND_) compared with DC alone (Supplementary Fig. 7a). Depletion of *TLR4* and *TRAF6* with CRISPR-Cas9 system (Supplementary Fig. 7b) generated *TLR4*^*−/−*^ and *TRAF6*^*−/−*^ DCs. The knockouts obliterated upregulation of MHC I/II, co-stimulatory molecules (Supplementary Fig. 7c, d) and secreted proinflammatory cytokines TNF-α and IL-12^12^ (Supplementary Fig. 7e) conferred by N1ND (TEX-N1ND vs. TEX) but did not affect upregulation associated with TEX (TEX vs. PBS controls). This suggests that TLR4 signaling pathway contributes to N1ND-mediated augmentation of DC activation, but not initial DC activation by TEX. The results firmly link TLR4 signaling to N1ND-mediated DC activation.

### N1ND-painted patient serum exosomes augment DC immunity

**Fig. 7:**
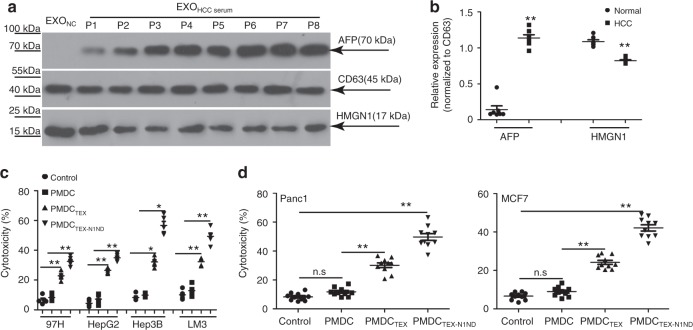
TEX-N1ND augmented human DC immunity. **a** Western blot analysis for detecting the levels of HMGN1 and AFP expression in serum exosomes derived from normal healthy volunteers and HCC patients. Total protein (20 μg) was loaded and CD63 was used as an exosomal marker protein. This experiment was repeated once (two in total). **b** Quantitative analysis of levels of HMGN1 (two-tailed *t* test) and AFP (two-tailed Mann-Whitney *U* test) in serum exosomes derived from normal healthy volunteers (*n* = 7) and HCC patients (*n* = 8). **c** Cytolysis rate against different human HCC cells with PMDC_TEX-N1ND_ and PMDC_TEX_. TEXs were derived from HCC patients’ serum. PMDC means peripheral mononuclear-derived dendritic cell. A LDH-releasing cytotoxic assay was performed to measure the cytolysis efficiency of effector lymphocytes activated by PMDC_TEX-N1ND_ or PMDC_TEX_, respectively (*n* = 5; one-way ANOVA post hoc Student–Newman–Keuls test except for Hep3B in which one-way ANOVA on ranks was used). Control refers to human PBMC-derived lymphocytes. **d** Cytolysis rate against human pancreatic and breast cancer cells with PMDC_TEX-N1ND_ and PMDC_TEX_. TEXs were derived from pancreatic or breast cancer patients’ serum, respectively (*n* = 10; one-way ANOVA on ranks). These experiments were repeated once (two in total). Data are presented as means ± s.e.m. (*p < 0.05; **p < 0.001; n.s not significant). Source data are provided as a Source Data file.

It was reported that HMGN1 expression levels are inversely correlated to clinical stage of tumor in cancer patients such as breast and non-small cell lung carcinoma^28^. HCC patient tumors expressed less HMGN1 than para-tumoral tissues, which was inversely correlated to the expression of HCC-specific antigen AFP (Supplementary Fig. 8a). Consistently, HCC patient serum exosomes contained significantly less HMGN1 than exosomes from normal volunteers’ serum (Fig. 7a, b), whereas AFP was up to eightfold higher (Fig. 7b). To determine the applicability of TEX-N1ND in the human setting, we loaded N1ND-modified exosomes, derived from HCC patients’ serum (TEX-N1ND), into human DCs derived from healthy volunteers’ PBMCs (PMDC_TEX-N1ND_). Allogeneic human lymphocytes activated by PMDC_TEX-N1ND_ resulted in significantly higher tumor-specific cytolysis rates against different human HCC cells than counterparts activated by PMDC_TEX_ or PMDC and unprimed lymphocytes irrespective of the origin of HCC cells (Figs. 7c and Supplementary Fig. 8b). Likewise, HMGN1 expression was reduced in exosomes from pancreatic and breast cancer patients’ serum compared with normal volunteers (Supplementary Fig. 8c, d). To explore the general applicability of N1ND as an immunoadjuvant in the clinic, we painted exosomes from pancreatic and breast cancer patients’ serum and loaded them into human PMDCs (PMDC_TEX-N1ND_). Strikingly, significantly higher cytolysis rates against pancreatic and breast cancer patient cells were detected in lymphocytes stimulated by PMDC_TEX-N1ND_ compared with corresponding PMDC_TEX_ or unprimed lymphocytes (Fig. 7d). The data demonstrate that N1ND can augment human DC immunogenicity in vitro in the presence of tumor-specific antigen and thus provide an avenue for improving DC vaccines.

## Discussion

It remains challenging to induce robust, durable antitumor immune response capable of markedly prolonging patient survival against large, poorly immunogenic tumors. Although DC vaccines show promises in the clinic with great safety profiles, limited clinical success has been achieved in treating large established tumors in either mice or human. Immunoadjuvants, particularly endogenous ones such as HMGN1, likely present fewer nonspecific immunological side effects than exogenous adjuvants such as bacterial lipopolysaccharides. Therefore, HMGN1 has been extensively investigated in combination with other chemotherapeutic agents and activators for other TLR pathways or checkpoint inhibitors^13–15^, but the effective delivery of HMGN1 to tumors has confounded researchers. Here we described a simple and effective approach to load the potent N1ND immunoadjuvant on TEXs via an exosomal anchor peptide (TEX-N1ND), resulting in efficient delivery of N1ND to DCs together with TAAs. TEX-N1ND dramatically enhances DCs’ antitumor immunity by improving DC activation, homing capacity to lymph nodes and the generation and amplification of long-lasting memory T cells. Memory T cells consequently contribute to robust and persistent tumor regression in mice bearing large established tumors. Strikingly, the amplified immunogenicity of DCs triggered by TEX-N1ND yielded prolonged tumor regression and persistent antitumor immune response, and also reshaped the immune and tumor microenvironment in mice with poorly immunogenic orthotopic large HCCs. This is particularly important for HCC as it is notorious for highly immunosuppressive tumor microenvironment which facilitates tumor evasion of the immune system^29^. Notably, TEX-N1ND could trigger a greater antitumor immunity of DCs if only CD63-positive TEXs were used. However, given the cost and efforts required for doing so, it might not be a worthwhile trade-off. Furthermore, TEX-N1ND enabled the augmentation of human DCs’ immunogenicity irrespective of the endogenous expression level of HMGN1 in or origins of TEXs, demonstrating the clinical and general applicability of N1ND as an immunoadjuvant in DC-based immunotherapy. This is the first study, to our knowledge, to demonstrate the potency of N1ND as an immunoadjuvant and a simple approach to load TEXs with various cargoes for delivery and thus our study provides a platform to augment antitumor immunity induced by DC vaccines.

In our current study, although tumor growth was significantly inhibited with DC_TEX-N1ND_ in different mouse models bearing large tumor loads, all the mice still succumbed to tumor, underlining the tenacity of large established tumors at overcoming current therapeutics. Nevertheless, long-lasting antitumor immunity was established in both subcutaneous and orthotopic tumor mouse models, which remarkably prevented tumor progression and lung metastasis, two crucial outcomes that can lead to prolonged quality-adjusted survival in patients with a large tumor burden. The reduction in tumor size may also allow surgical intervention that would have been previously impossible. Further optimization including the delivery route and dosing regimens and long-term efficacy will be investigated in our future studies. Notably, both DC cell line (DC2.4) and the more clinically acceptable bone marrow (BM)-derived primary DCs were tested in our study and similar cellular uptake efficiency and immunogenicity were observed when DCs were pulsed with TEX-N1ND under an identical protocol, indicating the source of DCs has no impact on the potency of N1ND and TEXs. However, considering that the low yield of BMDC limits its scale-up, particularly for in vivo studies, we used DC cell line for subsequent orthotopic studies. Perhaps as a positive, we believe that we are moderately successful at shifting the bottleneck of DC immunotherapy from the lack of immunostimulation to production of sufficient numbers of DC for therapy, which can be considered a significant progress in this field.

HMGN1 was chosen in our study as it can recruit and activate not only DCs but also NK cells, neutrophils, and monocytes/macrophages^12,13,30^. The truncated N1ND fragment painted on TEX as expected was effective at DC activation, which consequently boosted CD8^+^ T cells. Also significantly increased numbers of memory T cells were induced by DC_TEX-N1ND_, which largely contribute to the long-lasting antitumor immunity. Notably, the increase in memory T cells induced by DC_TEX-N1ND_ over DC_TEX_ in blood was mainly in the number of effector memory T cells rather than central memory T cells as evidenced by significantly elevated numbers of effector memory T cells and only a marginal increase in central memory T cells from DC_TEX-N1ND_-treated group compared with DC_TEX_. As expected, NK cells significantly increased in blood and tumor tissues from mice with large established tumors in three different tumor mouse models (Supplementary Fig. 9a), suggesting that there could be other non-T cell innate immune effects related to the long-lasting antitumor immunity elicited by TEX-N1ND^12^. Nevertheless, T cell-specific immune responses are primarily responsible for the observed antitumor effect as there was no significant tumor inhibition detected in subcutaneous HCC-bearing thymus-deficient but NK-intact nude mice when treated with BMDC_TEX-N1ND_ (Supplementary Fig. 9b, c).

Interestingly, HMGN1 was reported to decline in advanced stages of breast and non-small cell lung cancer patients^28^, and is also likely to be the case for HCC and pancreatic cancer. We have indeed observed that HMGN1 expression is lowered in HCC tumor than para-tumoral tissues. In this study, we show that TEX-N1ND is effective irrespective of the endogenous HMGN1 expression in tumors and it seems that it works better in tumors with low HMGN1 expression, which is loosely correlated to staging of cancer. Furthermore, this DC vaccine can also be used in large poorly immunogenic tumors. Thus, we are hopeful that the patients who are going to benefit the most from this treatment modality are those with advanced cancers that do not respond to other therapies. Worthy of mentioning, a uniqueness of our study is that we demonstrated the ability to use cancer exosomes to deliver endogenous tumor antigens and exogenous cargoes (such as N1ND) by painting with CP05, and thus simplify the process of developing improved DC vaccines.

A difficulty to test our system in autochthonous HCC mouse models bearing large tumor burdens is that it is hard to control tumor size as multinodular, dispersed, and nonuniform tumors formed in carcinogen-induced autochthonous HCC mice^31^. In order to closely evaluate the immunosuppressive nature of local tumor immune microenvironment manifested in late-stage HCC patients, we transplanted fewer tumor tissues to allow the establishment of immunosuppressive microenvironment in orthotopic HCC mice as demonstrated by significantly increased numbers of immunosuppressive Tregs and elevated levels of TGF-β in blood and tumor tissues from day-21 orthotopic HCC mice compared with normal controls (Supplementary Fig. 3a). Therefore, as a proof-of-concept study, orthotopic HCC transplantation models were used. However, further long-term investigation of DC_TEX-N1ND_ will be undertaken in diethylnitrosamine-induced autochthonous HCC models after optimization of treatment doses and kinetics.

Taken together, our study demonstrates a simple and direct approach to efficiently load cargoes for TEX, as well as the potency of N1ND in augmenting DC immunogenicity by promoting DC activation, homing capacity to lymph nodes and thus accelerating the generation of long-lasting memory T cells. This in turn contributes to the robust and persistent antitumor immunity in large established tumors in mice and human cell in vitro. Our study provides a platform for amplifying the antitumor immunity of DC vaccines and thus accelerates the development of DC-based immunotherapy.

## Methods

### Mice

*C57BL/6* wild-type (H-2^b^), *BALB/C* mice (H-2^d^), and thymus-deficient *BALB/C* nude mice (6–8-week old) were used in all experiments (five mice were used in each group for ectopic or orthotopic studies unless otherwise specified). Mice were housed under specific pathogen-free conditions in a temperature- and humidity-controlled room. All the animal experiments were carried out in the animal unit, Tianjin Medical University (Tianjin, China) according to procedures authorized and specifically approved by the institutional ethical committee (Permit Number: SYXK 2009–0001). Mice were sacrificed by CO_2_ inhalation at desired time-points, and tissues were either snap-frozen in liquid nitrogen-cooled isopentane or fixed with Bouin’s solution (Sigma, USA) and embedded with paraffin for histological study.

### Cell lines

Murine DC line DC2.4 (referred to DC) (H-2^b^) (kindly provided by Dr De Yang, Center for Cancer Research, NIH, USA) was used for murine orthotopic HCC studies^32^. Briefly, DC cells were cultured in Dulbecco’s modified Eagle’s medium (DMEM) with 1% antibiotics, 1% glutamine (Gln), 50-μM β-mercaptoethanol, and 10% depleted fetal bovine serum (FBS, Hyclone, USA), obtained by centrifugation at 100,000 *g* for 1 h to remove possible FBS-containing exosomes. Murine HCC cell line Hepa1-6 (H-2^b^) was purchased from Boster Biological Technology Ltd (Wuhan, China) and cultured in DMEM with 2-mM Gln and 10% FBS as per manufacturer’s instructions. Murine pancreatic cancer cell line (Panc02, H-2^b^), Lewis lung cancer cell line (LLC1, H-2^b^), breast cancer cell line (4T1, H-2^d^), cervical cancer cell (Hela), and lymphoma cell (EL4) were kept in house and cultured as previously described^33,34^. Human HCC cell lines including HepG2 and Hep3B (purchased from ATCC biobank) and MHCC-97H (purchased from Shanghai Institute for Biological Sciences, Chinese Academy of Sciences) and LM3 (purchased from BeNa Culture Collection, Beijing, China) and cultured as per manufacturer’s instructions^35^. Human pancreatic cancer cell line (Panc1) and breast cancer cell line (MCF7) were purchased from ATCC biobank and cultured as per manufacturer’s instructions. Briefly, cells were grown at 37 °C in 5% CO_2_ in DMEM supplemented with 10% FBS obtained by centrifugation at 100,000 *g* for 1 h to remove possible FBS-containing exosomes, and 1% penicillin and streptomycin.

### Peptides

N1ND (HMGN1^1–52^)^12^ and N1ND-CP05 were synthesized as a single fusion peptide via peptide bond without spacer by Chinapeptide (Suzhou, China) with 96% of purity and labeled with AF680 by Prof. Qibing Zhou’s lab (Huazhong University of Science and Technology, Wuhan, China).

### Establishment of ectopic and orthotopic tumor mouse models

Subcutaneous tumor mouse models bearing large tumor loads were established by subcutaneous injection of 0.1 ml PBS containing Hepa1-6 cells (2 × 10^6^), Panc02 (2 × 10^6^), and 4T1 (2 × 10^6^) into left axilla of *C57BL/6*, *BALB/C,* or thymus-deficient *BALB/C* nude mice (5 × 10^5^ of Hepa1-6 cells) (*n* = 5, 6–8-week old) and allowed to grow for 14 days or 7 days (for nude mice). For the establishment of orthotopic HCC mice, subcutaneous tumors with a longitudinal diameter of 1 cm were peeled from subcutaneous mouse models after schedule 1 killing. Tumor tissues were washed in D-hanks buffer. Necrotic tissues were removed from tumors and tumor tissues were cut into about 1 mm^3^ pieces. Two to three tumor pieces were implanted in the left lobe of liver in the recipient mice under anesthesia. The length (*L*) and width (*W*) of tumors were measured with a caliper. Tumor size was calculated by the formula: (*L* × *W*^2^)/2^18^.

### Isolation of murine DCs, human DCs, and lymphocytes

For murine in vitro studies, BM-derived DCs were generated as previously reported^36^. BM progenitors (1 × 10^6^ cells per ml) isolated from BMs of *C57BL/6* or *BALB/C* mice were cultured in Roswell Park Memorial Institute (RPMI) 1640 medium plus 10% FBS, 1% P/S (100 U ml^−1^ penicillin and 100 μg ml^−1^ streptomycin), murine granulocyte-macrophage colony-stimulating factor (GM-CSF) (200 U ml^−1^) (PeproTech, USA) and interleukin-4 (IL-4) (100 U ml^−1^) (PeproTech, USA), and 50 μmol l^−1^ β-mercaptoethanol at 37 °C in a humidified incubator with 5% CO_2_ for 5–7 days to generate immature DCs. Subsequently, immature DCs (1 × 10^6^ cells per ml) were incubated in fresh culture medium in the absence or presence of TEX or N1ND-CP05 modified TEX (TEX-N1ND) for 48 h at 37 °C in a humidified incubator with 5% CO_2_ prior to functional and phenotypical analysis. For human in vitro studies, human peripheral blood from healthy volunteers was purchased from Tianjin Blood Center (Tianjin, China). Peripheral blood mononuclear cells (PBMCs) were isolated with human Lymphoprep solution (Axis-shield PoC AS, Oslo, Norway) as per manufacturer’s instructions, then cells were seeded in 10-cm petri-dish with 10 ml RPMI 1640 medium plus 10% FBS and incubated for 2 h to allow cells to adhere to the surface. Adherent cells were harvested and induced to form immature DCs by culturing in RPMI 1640 medium containing 20% FBS, 120 ng ml^−1^ recombinant human GM-CSF (PeproTech, USA) and 60 ng ml^−1^ recombinant human IL-4 (PeproTech, USA) for 5 days. Non-adherent cells were recovered and cultured in DMEM/F-12 medium containing 10% FBS for 7 days and were used as mixed population of human lymphocytes.

### Preparation of exosomes

Cell culture medium was sequentially centrifuged at 1000 *g* for 10 min, followed by 10,000 *g* for 30 min. The supernatant was collected and filtered with a 0.22-μm filter (Millex, Germany), followed by ultracentrifugation at 100,000 *g* for 1 h to pellet exosomes. Exosome pellets were washed in a large volume of PBS and recovered by centrifugation at 100,000 *g* for 1 h. The total protein concentration of exosomes was quantified by Bradford assay (Sangon Biotech, USA). For human serum exosome isolation approved by the institutional review board, human serum from healthy volunteers or cancer patients was obtained from Tianjin Blood Center (Tianjin, China) or from biobank of Tianjin Medical University Cancer Institute and Hospital (Tianjin, China), with the approval of the hospital ethic committee and Tianjin science and technology commission (Permit number (2016) 621). Serum was centrifuged at 3000 *g*, 6,000 *g*, and 10,000 *g* twice for 30 min each time to remove cell debris, followed by filtration with 0.22-μm filter (Millex, Germany). The supernatant was transferred into a fresh tube and pelleted by ultracentrifugation (Beckman Optimal-100 XP, Beckman Coulter, Germany) at 100,000 *g* for 1 h and further purified by sucrose density gradient centrifugation. Exosomes were layered on a linear sucrose gradient (0.25 M, 0.45 M, 0.65 M, 0.85 M, 1.05 M, 1.25 M, 1.45 M, 1.65 M, and 1.85 M sucrose, Sigma, China). The gradients were centrifuged for 18 h at 100,000 *g* at 4 °C. Six fractions from 0.65 to 1.65 M sucrose gradients were collected and ultracentrifuged at 100,000 *g* for 1 h at 4 °C to pellet exosomes. The exosome pellets were dissolved in PBS and the total protein concentration of exosomes was quantified by the Bradford assay (Sangon Biotech, USA).

### Characterization of exosomes

Exosomal morphology was visualized using a high-resolution transmission electron microscope (TEM, Hitachi HT7700, Tokyo, Japan)^19^. Briefly, the resuspended exosomes were diluted into PBS (1 μg μl^−1^) and mixed with an equal volume of 4% paraformaldehyde. Exosomes were adsorbed onto a glow-discharged, carbon-coated formvar film attached to a metal specimen grid. Excess solution was blotted off and the grid was immersed with a small drop (50 μl) of 1% glutaraldehyde for 5 min followed by washing with 100 μl distilled water for eight times (2 min each time). Subsequently, the grid was transferred to 50 μl uranyl-oxalate solution (pH7.0) for 5 min and then 50 μl methyl cellulose-uranyl acetate (100 μl 4% uranyl acetate and 900 μl 2% methyl cellulose) for 10 min on ice. The excess solution was blotted off and the sample was dried and examined with TEM.

### Antigen-specific assay

Subcutaneous HCC mice were established by subcutaneous injection of 0.1 ml PBS containing Hepa1-6 cells (5 × 10^5^) into left axilla of *C57BL/6* mice (*n* = 5, 6–8-week old) and allowed to grow for 7 days, followed by intravenous injection of DC_TEX(Hepa)_, DC_TEX(Hepa)-N1ND_, DC_TEX(Panc)-N1ND_, and DC _TEX(4T1)-N1ND_ at the dose of 2 × 10^6^ cells once per week for 3 weeks. Tumor and splenocytes were harvested 48 h after last immunization for measurement. Splenocytes (5 × 10^5^) per well were plated in 48-well plates and cultured in fresh T lymphocyte medium containing AFP212 (40 μg ml^−1^), a known murine AFP epitope^20^ (purchased from ChinaPeptides Co, Ltd, China), or without peptides for 72 h. Levels of IFN-γ and IL-2 were detected in supernatant of activated splenocytes per the manufacturer’s instructions. For the tetramer staining, generation of AFP-specific tetramer was performed as per manufacturer’s instructions (the QuickSwitchTM Quant H-2K^b^ tetramer kit; TB-7400-K1, MBL, USA) with AFP212 (10 μM). Splenocytes from different treatment groups were treated with 50 nM dasatinib (HY-10181, MCE, USA) for 30 min at 37 °C, followed by washing with washing buffer and stained with AFP-H-2K^b^-tetramer-PE (2 μg ml^−1^) for 60 min at 4 °C. Subsequently, tetramer stained splenocytes were counterstained with FITC-anti -mouse CD3e (eBioscience, USA) and APC-anti-mouse-CD8α (Biolegend, USA). The stained cells were subjected to flow cytometric analysis with BD LSRFortessa and analyzed by FlowJo software (FlowJo, LLC).

### MRI assessment

The magnetic resonance images of orthotopic HCC mice were acquired using T2 propeller sequence with the following parameters: slice thickness of 1.0 mm, slice spacing of 0.5 mm, TR/TE of 3494/70.7 ms, matrix of 256 × 160, and FOV of 8 × 8 cm (3.0 Tesla MR scanner, Signa Excite HDx; GE healthcare, Milwaukee, WI, USA) with a small animal coil in Tianjin Medical University General Hospital. During the examination, mice were anesthetized with pentobarbital sodium and fixed to minimize body motion, respiration rate was monitored, and body temperature was maintained to be at 37 °C using warm airflow. The tumor size was calculated based on MRI images for 3, 5, and 7 weeks in orthotopic HCC mice bearing large established tumor. Each mouse was marked and imaged with MRI at different time-points.

### Peptide binding and cellular uptake assay

To measure the percentage of CD63-positive exosomes in total exosomes, cellmask-labeled (Life Technologies, USA) exosomes (5 μg) were incubated with phycoerythrin (PE)-cy7 anti-mouse CD63 antibody (1:500; eBioscience, USA) or PE-cy7 rat immunoglobulin G2a (IgG2a) isotype control (1:500; eBioscience, USA) in 4% BSA for 30 min at 4 °C, followed by 1:10 dilution with PBS, and washed with PBS and filtered to remove unbound peptides before flow cytometry (BD LSRFortessa™ cell analyzer, USA). To measure the binding affinity of N1ND-CP05 or N1ND on exosomes, PKH67-labeled (Sigma, USA) exosomes (10 μg) derived from Hepa1-6, Panc02, or 4T1 were incubated with AF680-labeled peptides (20 μg) overnight at 4 °C, followed by washing with PBS for five times in 2-ml ultracentrifuge tubes and filtration with 100-kDa diafiltration tube (Millipore, USA) to remove unbound peptides. Subsequently, peptide-exosome complexes (30 μg) were measured by flow cytometry directly^19^. Briefly, to validate the flow cytometry assay for exosomes, we included (1) a buffer control to eliminate the background noise; (2) the resuspended pellet from a 100,000 *g* ultracentrifugation of cell culture medium to evaluate the background noise; (3) 100 and 200 nm reference beads to define the target exosome populations. Exosomes alone were used as negative controls for gating. To test the uptake of different peptide/ exosome complexes in murine BMDCs, PKH67-labeled exosomes (40 μg) derived from Hepa1-6, Panc02, or 4T1 were pre-incubated with AF680-labeled peptides (80 μg) overnight at 4 °C, then added into BMDCs derived from either *C57BL/6* or *BALB/C* mice (on day 7 after differentiation) at 37 °C in 5% CO_2_. Cells were analyzed with flow cytometry (BD LSRFortessa™ cell analyzer, USA) at 24 h after incubation. For measurement of cellular localization, PKH67-labeled exosomes (40 μg) derived from Hepa1-6 cells were pre-incubated with AF680-labeled peptides (80 μg) overnight at 4 °C, then added into DCs (DC2.4) at 37 °C in 5% CO_2_ for 2 h or 24 h, followed by observation with confocal fluorescence microscope (Olympus FV1000, Olympus, Japan).

### Western blot

Protein extraction and western blot were carried out as previously described^18^. Various amounts of protein prepared from cell lysates and TEXs were loaded onto SDS-polyacrylamide gel (10%). The membrane was then washed and blocked with 5% skimmed milk and probed for 1 h with different primary antibodies including mouse monoclonal antibodies: Alix (1:200; Cell signaling technology, USA) and GAPDH (1:1000; MultiSciences Biotech Co., Ltd, China); and rabbit polyclonal antibodies: β-actin (1:2000; Cell signaling technology, USA), CD63 (1:200, Santa Cruz, USA), HMGN1 (1:1000; ProteinTech, USA), AFP (1:2000; Abcam, UK), TLR4 (1:1000; ProteinTech, USA), MyD88 (1:1000; Cell signaling, USA), and TRAF6 (1:5000; Abcam, UK), respectively. The bound primary antibodies were detected by peroxidase-conjugated goat anti-mouse, rabbit anti-mouse, or goat anti-rabbit IgGs (1:5000; Sigma, USA), respectively, and the ECL western blot analysis system (Millipore, Billerica, MA) was used. Uncropped images of all western blots can be found in Supplementary Fig. 10.

### Flow cytometry and intracellular staining

For the detection of murine DC or BMDC surface markers, DCs or BMDCs were washed in FACS buffer (PBS containing 0.5% BSA and 0.05% NaN3) and stained with FITC-anti-mouse IgG2b Isotype (1:500), PE-anti-mouse IgG2b Isotype (1:500), APC-anti-mouse IgG2b Isotype (1:500; Biolegend, USA), PE-cy7-anti-mouse IgG2b Isotype (1:500; eBioscience, USA), PE-cy7 anti-mouse CD63 (1:500; eBioscience, USA), FITC-anti-mouse MHC I (1:250; eBioscience, USA), FITC-anti-mouse MHC II (1:250; Biolegend, USA), FITC-anti-mouse CD80 (1:250; Abcam, UK), PE-anti-mouse CD83, and APC-anti-mouse CD86 (1:250; eBioscience, USA), and PE-cy5.5 anti-mouse CD11c (1:250; eBioscience, USA) on ice for 30 min prior to flow cytometry. For the detection of various subsets of leukocytes including NK cells, T lymphocytes and B cells in blood, lymphoid organs or tumor tissues, single-cell suspensions from different tissues were stained with FITC- or Percp-cy5.5-anti-mouse CD45 (1:500; Biolegend, USA), FITC-, PE-, or PE-cy7-anti-mouse CD3e (1:500; eBioscience, USA), PE-anti-mouse CD4 (1:500; eBioscience, USA), APC-anti-mouse-CD8α (1:500; Biolegend, USA), PE-anti-mouse CD11c (1:250; Biolegend, USA), BV421-anti-mouse-CD11b (1:500; Biolegend, USA), FITC-anti-mouse-B220/CD45R (1:500; eBioscience, USA), PE-cy7 anti-mouse-CD19 (1:500; Biolegend, USA), APC-anti-mouse-NK1.1 (1:500; eBioscience, USA), PE-cy7-anti-mouse-CD25 (1:500; eBioscience, USA), PE-anti-mouse-CD62L (1:500; eBioscience, USA), FITC-anti-mouse-CD44 (1:500; eBioscience, USA), PE-cy7 anti-mouse-CD127 (1:500; eBioscience, USA), and eFluro450-anti-mouse CCR7 (1:250; eBioscience, USA). For the detection of various subsets of lymphocytes derived from human PBMC, cells were stained with PE-anti-human CD45 (1:500; Biolegend, USA), PE-cy5-anti-human CD3e (1:500; Biolegend, USA), APC-anti-human CD4 (1:500; Biolegend, USA), FITC-anti-human-B220/CD45R (1:500; eBioscience, USA), FITC-anti-human-CD8α (1:500; Biolegend, USA), FITC-anti-human-CD56 (1:500; Biolegend, USA), and APC-anti-human CD19 (1:500; Biolegend, USA). Cells were washed with FACS buffer for three times after staining followed by flow cytometry (BD LSRFortessa™ cell analyzer, USA). All data were analyzed with the software FlowJo (Tree Star Inc., Ashland, OR, USA). Flow cytometry data were acquired by BD LSRFortessa, and analyzed by FlowJo software (FlowJo, LLC).

### Cytokine release and cytolysis assay

Murine DCs or BMDCs were pulsed with N1ND-modified Hepa1-6, Panc02, and 4T1 TEXs or exosomes from HCC, pancreatic cancer, and breast cancer patients’ serum and incubated for 48 h at the final concentration of 40 μg ml^−1^, respectively. Mixed splenic T lymphocytes were harvested from unimmunized *C57BL/6* or *BALB/C* mice and incubated with (BM) DC_TEX-N1ND_ or (BM)DC_TEX_ for 3 days. Levels of IFN-γ and IL-2 in supernatant were detected in activated T lymphocytes per the manufacturer’s instructions. For mouse in vitro study, IFN-γ and IL-2 ELISA kits were purchased from R&D systems and eBioscience (USA). For in vivo cytokine assay, mouse blood was harvested from orthotopic tumor-bearing mice 2 days after the last immunization and centrifuged at 3000 *g* for 30 min at room temperature, followed by analysis of IFN-γ (R&D systems, USA), IL-10, and TGF-β (eBioscience, USA), respectively. For human cytolysis assay, mixed population of lymphocytes derived from human PBMC were co-cultured with human DC activated by TEX-N1ND for 3 days. Subsequently, activated human lymphocytes were tested again different human cancer cells. The cytotoxicity detection kit (R&D systems, USA) was used to measure the cytolysis rate elicited by effector T cells against different tumor cells. For the cytolysis assay, 1 × 10^5^ effector T cells (E) were used to lyse against 1 × 10^4^ target cells (T) with an E:T ratio of 10:1. For other E:T ratios, the number of target cells remained the same while the number of effector T cells changed accordingly. The amount of lactate dehydrogenase (LDH) released from lysed target cells was used as an indicator for cytolysis. Cytolysis rate (%) was calculated based on the equation: cytotoxicity (%) = (((effector/target cell mix − effector cell control) − low control)/(high control − low control)) × 100 as per manufacturer’s instructions (Roche, CA, USA).

### Isolation of leukocytes from mouse blood and tissues

Blood from ectopic and orthotopic tumor-bearing mice treated with (BM)DC_TEX-N1ND_, (BM)DC_TEX_, (BM)DC, or PBS was collected with 1% heparin, followed by addition of equal volume of Lymphoprep™ (Stemcell Technologies, Canada) and lysis with the ammonium chloride-potassium (ACK) lysis buffer for 5 min at room temperature to generate lymphocyte suspensions. Isolation of T lymphocytes from mouse tumor tissues was adopted^37^. Briefly, tumor tissues were minced into small pieces with surgical scissors, gently forced through a 200-μm-gauge stainless steel mesh with a sterile syringe plunger and digested in collagenase type IV suspension (0.05 mg ml^−1^, Worthington Biochem. Corp., USA) for 40 min at 37 °C. The resulting suspension was filtered through the 70-μm cell strainer. The extract was centrifuged at 528 *g* for 10 min and the supernatant was removed. The mixture was resuspended with 10 ml 40% percoll (Pharmacia, Sweden) followed by centrifugation at 850 *g* for 30 min at 22 °C to remove the supernatant. Cell pellets were resuspended in ACK lysis buffer to remove red blood cells and the rest cells were stained with different fluorescence-labeled antibodies as described above.

### Immunohistochemistry and histological staining

To examine the presence of CD3^+^ T and Treg lymphocytes in tumor sections from orthotopic HCC mice treated with DC_TEX-N1ND_ or control groups, mouse tumor tissues were fixed in Bouin’s solution (Sigma, USA) and embedded with paraffin. CD3^+^ or Foxp3^+^ T lymphocytes were stained with rabbit anti-mouse polyclonal antibodies CD3 (1:2000; Novus, USA) or Foxp3 (1:2000; Abcam, UK), respectively, and detected by goat anti-rabbit secondary antibody (1:5000; Sigma, USA). Routine Hematoxylin and eosin (H&E) staining was used to examine the pulmonary metastasis of lung tissues from orthotopic HCC mouse models. Briefly, tissues were fixed in Bouin’s solution and embedded with paraffin followed by staining with hematoxylin and eosin. The slides were incubated in filtered 0.1% Mayer’s haematoxylin for 10 min for nucleus and ribosome staining. Afterwards, sections were rinsed in cool-running distilled H_2_O to remove excessive haematoxylin, and treated with 0.5% hydrochloric acid, and 1% sodium bicarbonate solution, respectively. After that, sections were incubated in 0.5% eosin (0.5 g dissolved in 100 ml of 95% EtOH) for 5 min for cytoplasm staining, and washed by distilled H_2_O. The sections were dehydrated in ingredients of 70, 80, 95, 100, 100% EtOH for 5 min each. Finally, sections were treated in xylene for 10 min and coverslips were mounted with neutral gum, followed by visualization with an inverted microscope (OLYMPUS, Tokyo, Japan).

### Tissue distribution

To examine the biodistribution DC_TEX-N1ND_ or DC_TEX_ in orthotopic *C57BL/6* HCC mice, PE-DiI-labeled (ThermoFisher, USA) DC_TEX-N1ND_, DC_TEX_, or DC (5 × 10^6^) were administered intravenously into orthotopic *C57/BL6* HCC mice for single injection. Liver, tumor, mesenteric lymph node, inguinal lymph node, kidney, and spleen were harvested 48 h after injection for imaging with IVIS spectrum (PE, Waltham, MA, USA).

### In vitro assay for T cell activation and proliferation

To examine the effect of DC_TEX-N1ND_ on memory and naive T cells, wild-type *C57BL/6* mice were immunized with DC_TEX_ (2 × 10^6^) intravenously for once per week for 3 weeks and splenocytes were harvested 4 weeks post vaccination. T cell subsets including CD8^+^CD44^high^CD62L^high/low^ memory and CD8^+^CD44^low^CD62L^high^ naive T cells were isolated with FACS sorting with BD FACSAriall (BD Biosciences, USA). Memory or naive T cells (3 × 10^5^) were then incubated with DC_TEX_ or DC_TEX-N1ND_ (3 × 10^4^) for 3 or 6 days in 96-well plates. On day 3 or 6 after co-incubation, each CD8^+^ T cell subset in treated samples was analyzed by flow cytometry.

### T cell adoptive transfer

*C57BL/6* mice were immunized with DC_TEX-N1ND_ (2 × 10^6^) once per week for 3 weeks. CD8^+^CD44^high^CD62L^high/low^ memory T cell subsets were harvested from spleen and lymph nodes of immunized mice 4 weeks after last immunization by flow cytometry sorting with BD FACSAriall (BD Biosciences, USA). CD8^+^CD44^high^CD62L^high/low^ memory T cells (5 × 10^6^) were injected intravenously into day-7-established orthotopic HCC mice for once. DC_TEX-N1ND_ was administered as a positive control with the same dosing regimen as used in the immunization protocol. The tumor volume was measure 2 weeks after treatment as described above.

### Generation of *TLR4/TRAF6*-deficient DC cell lines

DC2.4 were transfected with CRISPR/Cas9-expressing lentivirus (Lentiviral CRISPR Toolbox, Zhang lab, USA), followed by 2 μg ml^−1^ blasticidin selection for 1 week. Cas9-expressing DC2.4 cells were transfected with sgRNA-expressing lentivirus for 12 h, followed by 0.5 μg ml^−1^ puromycin selection for 2 weeks. sgRNAs targeting murine *TLR4* and *TRAF6* genes were designed with the CRISPR/Cas9 Target Online Predictor (crispr.cos.uni-heidelberg.de, University of Heidelberg, Germany)^38^. The *TLR4* gene target sgRNA sequences were 5′-CACCGAGAGTCCTAGCCAGGAGCCA-3′, 5′-AAACTGGCTCCTGGCTAGGACTCTC-3′; the *TRAF6* gene target sgRNA sequences were 5′-CACCGTTAAACTGTGAGAACAGCTG-3′ and 5′-AAACCAGC TGTTCTCACAGTTTAAC-3′, respectively. These sgRNAs were cloned into a LentiGuide-puro vector at BsmBI site as previously reported^39^.

### Statistical analysis

All data are presented as means ± s.e.m. Statistical differences between treated and control groups were evaluated by SigmaStat (Systat Software). Both parametric and nonparametric analyses were applied as specified in figure legends. Sample size was determined by G*Power 3.1.7 (Power analysis and Sample size). Significance was determined based in *p* < 0.05.

### Reporting summary

Further information on research design is available in the Nature Research Reporting Summary linked to this article.

## Supplementary information

Supplementary Information

Reporting Summary

## Data Availability

The source data underlying Figs. 1a, e–h, 2a–g, 3b, d, e, 4a, b, d–f, 5a–j, 6a–g, 7a–d and Supplementary Figs. 1a, b, 2d–f, 3a, d, e, 4a, b, 5b–e, 7a, b, d, e, 8a, c, d, 9a and c are provided as a Source Data file. All the other data supporting the findings of this study are available within the article and its supplementary information files and from the corresponding author upon reasonable request.

## Source Data

Source Data
