# Supplementary material for: Alarmin-painted exosomes elicit persistent antitumor immunity in large established tumors in mice
Source: Nat Commun. 2020 Apr 14;11:1790. doi: 10.1038/s41467-020-15569-2 (PMC7156382; doi:10.1038/s41467-020-15569-2)

Supplementary Information

Alarmin-painted exosomes elicit persistent antitumor immunity in large established tumors in mice

Zuo et al.

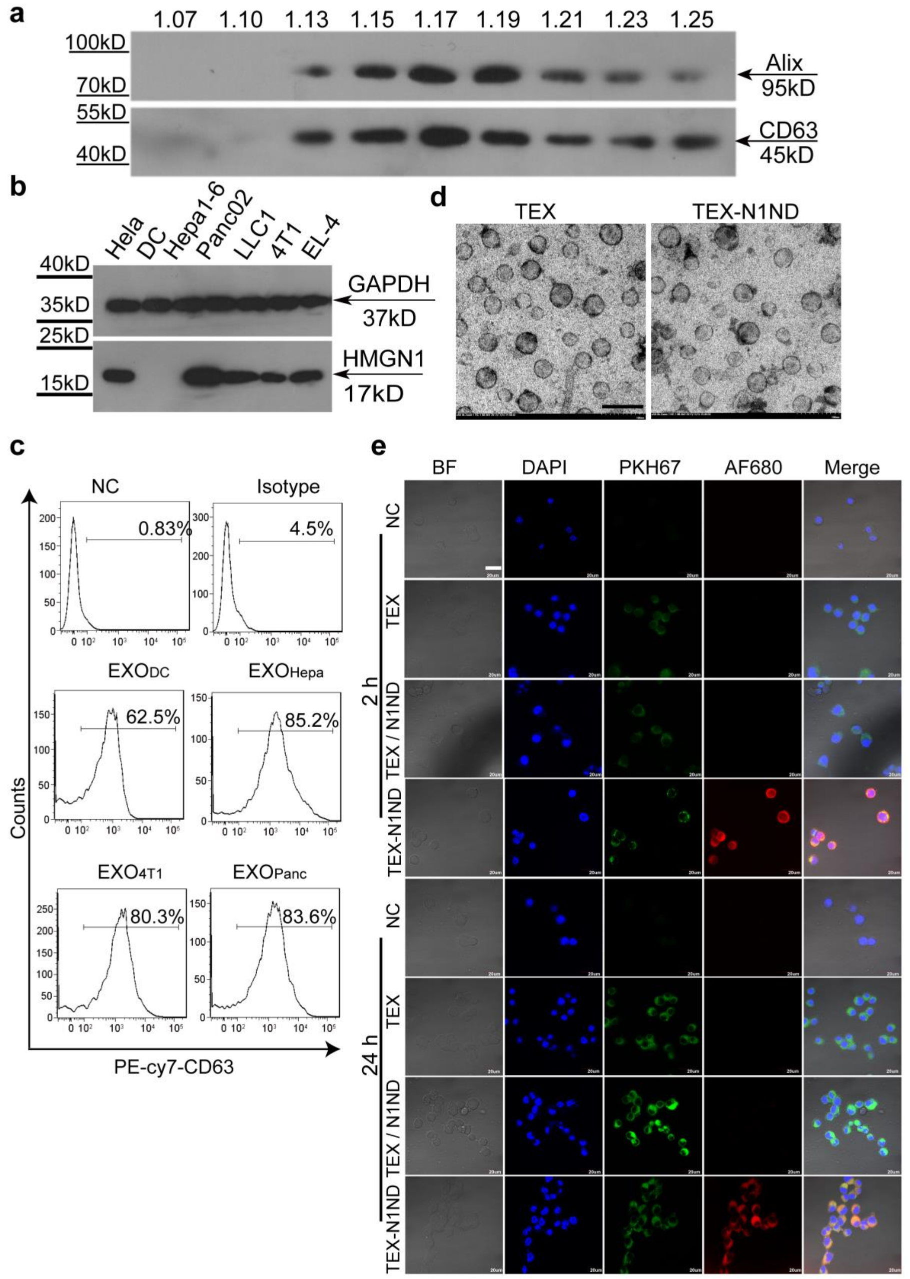

Supplementary Figure 1. ***In vitro*** **characterization of exosomes from murine Hepa1-6 cells (TEXs).** (**a**) Western blot analysis of buoyant density for exosomes after gradient sucrose centrifugation. Total exosomal protein (20 μg) was loaded. Alix and CD63 were used as exosomal markers. (**b**) Western blot analysis to determine the expression of HMGN1 on different cells. Total protein (50 μg) was loaded and GAPDH was used as a loading control. This experiment was repeated once (two in total). (**c**) Quantitative analysis of CD63 expression on exosomal surface with flow cytometry. EXO means exosomes. (**d**) Transmission electron micrographic image (TEM) of N1ND-CP05 modified TEXs (scale bar=100 nm). This experiment was repeated twice (three in total) (**e**) Cellular uptake of TEX loaded with N1ND-CP05 or N1ND alone (40 μg per ml) in DCs (DC2.4) at 2 h or 24 h after incubation (scale bar =20 μm). Hepa1-6 derived exosomes were labelled with PKH67 and peptide was labelled with AF680. This experiment was repeated once (two in total). Source data are provided as a Source Data file.

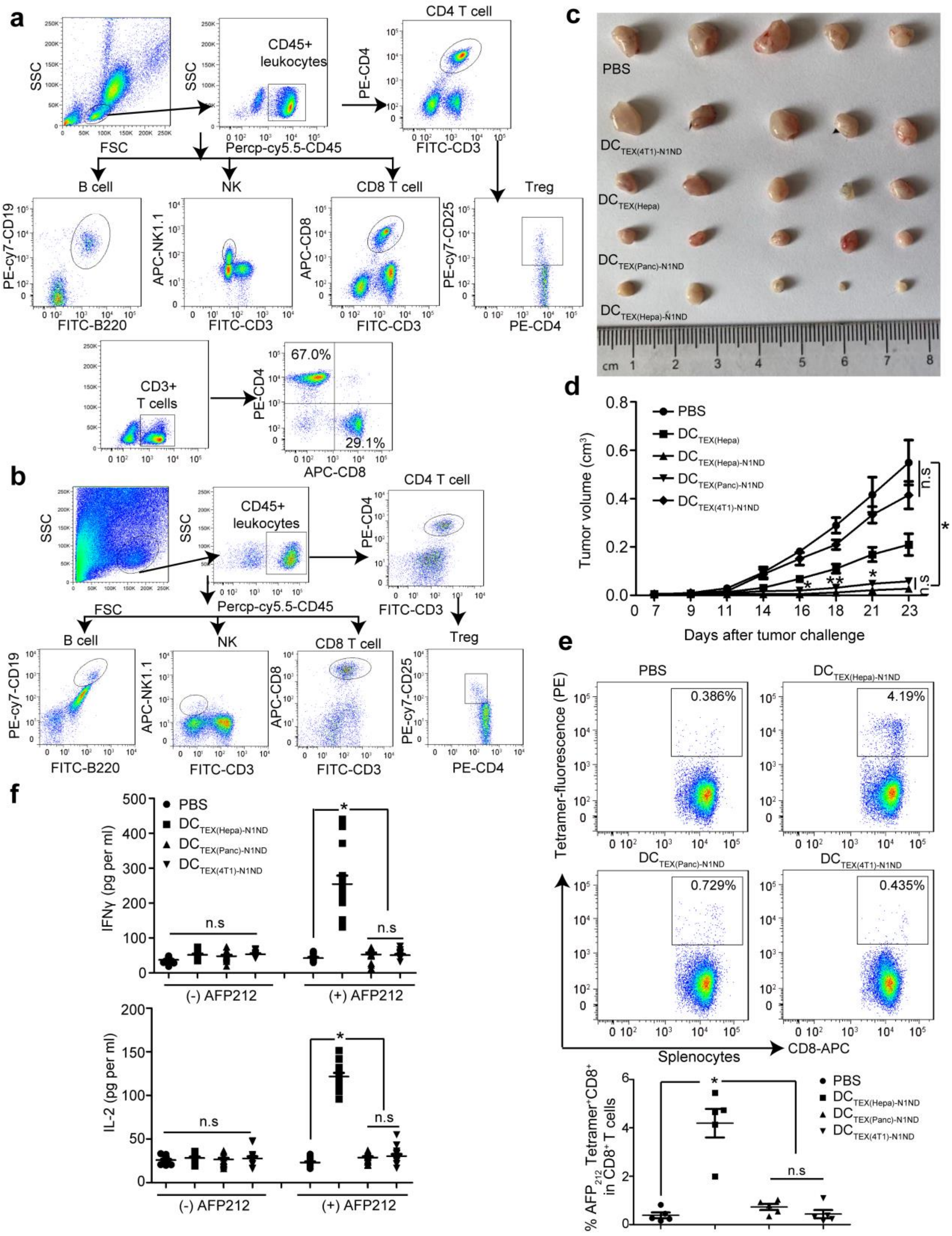

Supplementary Figure 2. Gating strategy used for cell sorting and antigen-specific evaluation of N1ND modified TEXs in subcutaneous HCC mice. Gating strategy for the characterization of different immune cells from mouse blood (**a**) presented on Fig. 2b, 2f, 4a, 4b, supplementary Fig. 3a, 9a or tumor (**b**) presented on Fig. 2c, 2g, 4a, 4b, supplementary Fig. 3a and 9a. Representative tumor images (**c**) or measurement of tumor volume (**d**) of day-7 established subcutaneous HCC mice treated with $DC_{TEX\text{-}N1ND}$ or DC_{TEX} ($2x10^6$ cells once per week for 3 weeks) at day16 (one-way ANOVA on ranks), day 18 and 21 (one-way ANOVA post hoc Student-Newman-Keuls test), and day 23 (one-way ANOVA on ranks) (n=5). (**e**) Flow

cytometric analysis of AFP-specific $CD8^+$ T cells in splenocytes from subcutaneous HCC mice treated with $DC_{TEX\text{-}N1ND}$ or DC_{TEX} ($2x10^6$ cells once per week for 3 weeks) 48 h after last injection (n=5; one-way ANOVA on ranks). (**f**) Measurement of IFN-γ and IL-2 in culture supernatants of splenocytes isolated from $DC_{TEX\text{-}N1ND}$-treated subcutaneous HCC mice, followed by stimulation with or without AFP212 for 72 h (n=5; one-way ANOVA on ranks). Triplicates were tested for each sample. $DC_{TEX(Hepa)\text{-}N1ND}$ or $DC_{TEX(Panc)\text{-}N1ND}$ refers to DCs loaded with N1ND modified exosomes derived from Hepa1-6 or Panc02 cells, respectively. Data are presented as means ± s.e.m. (*p<0.05; **p<0.001; n.s = not significant). Source data are provided as a Source Data file.

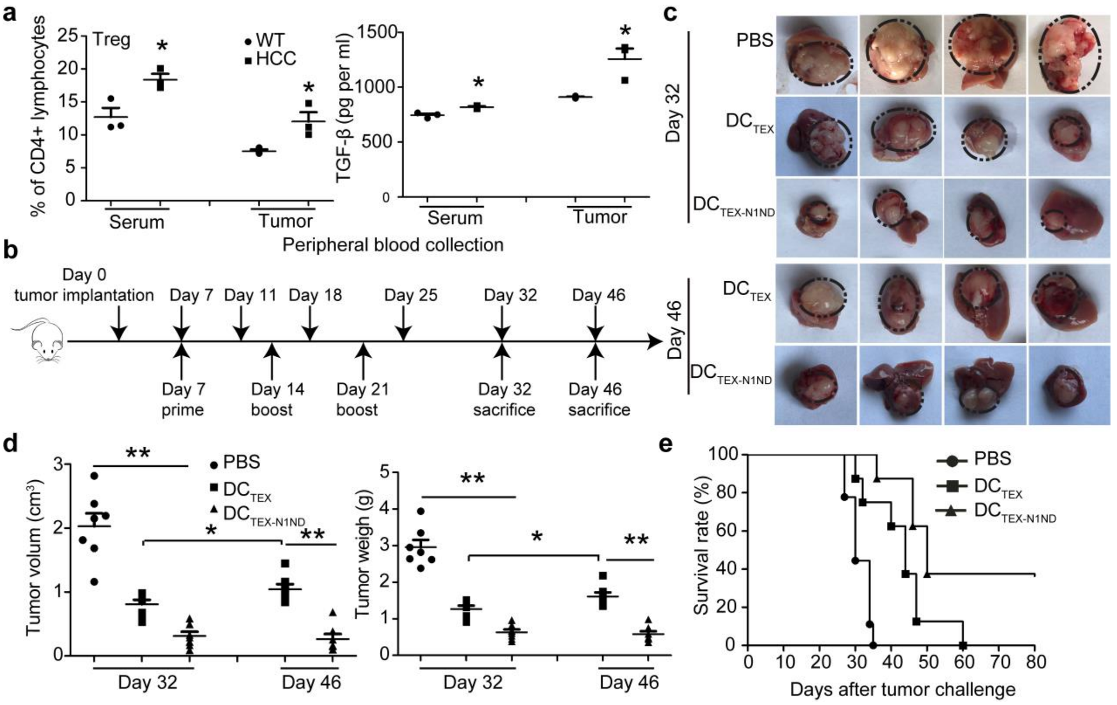

Supplementary Figure 3. Measurement of immune microenvironment in day-21 orthotopic HCC mice and systemic evaluation of $DC_{TEX-N1ND}$ in day-7 orthotopic *C57BL/6* HCC mice. (**a**) Flow cytometric analysis of immunosuppressive Tregs or ELISA for TGF-β cytokines in blood and tumor tissues from day-21 orthotopic HCC mice (n=3; two-tailed t test). WT means wild-type mice. (**b**) Schematic diagram for the dosing regimen of $DC_{TEX-N1ND}$ in day-7 orthotopic *C57BL/6* HCC mice therapeutically. Day-7 orthotopic HCC mice were treated intravenously with $DC_{TEX-N1ND}$, DC_{TEX} ($2x10^6$ once per week for 3 weeks) and PBS, respectively. Tissues were harvested on day 32 and 46 after tumor challenge. (**c**) Representative tumor images from different treatment groups on day 32 and 46. (**d**) Analysis of tumor volume and weight from day-7 orthotopic HCC mice treated with PBS, DC_{TEX} or $DC_{TEX-N1ND}$ on day 32 (one-way ANOVA on ranks) and 46 (two-tailed t test) (n=7). (**e**) Survival rate of day-7 orthotopic HCC mice treated with PBS (n=9), DC_{TEX} (n=8) or $DC_{TEX-N1ND}$ (n=8), respectively. Data are presented as means ± s.e.m. (*$p<0.05$; **$p<0.001$). Source data are provided as a Source Data file.

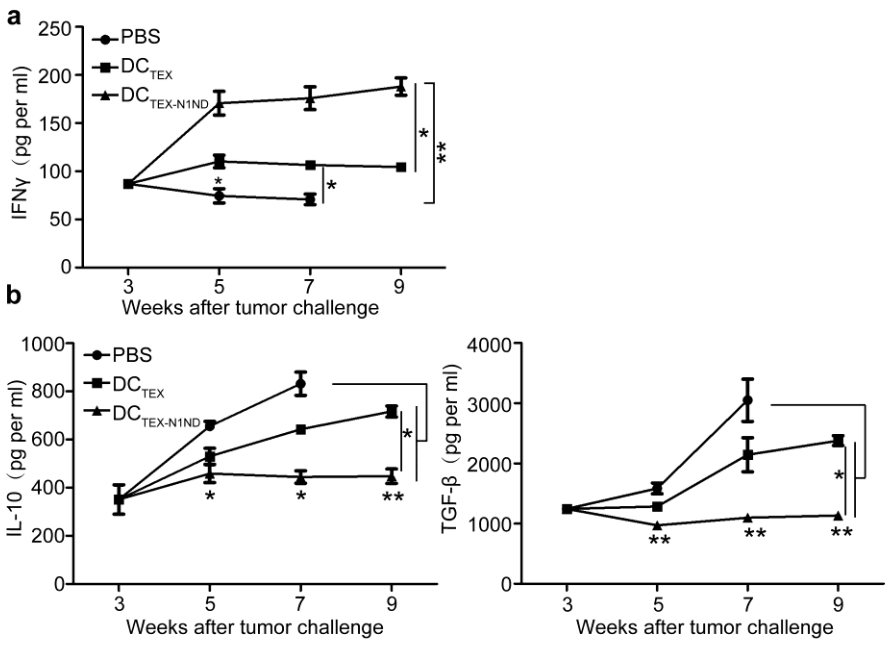

Supplementary Figure 4. Measurement of immune microenvironment in blood from day-21 orthotopic *C57BL/6* HCC mice treated with DC$_{\text{TEX-N1ND}}$ at different time-points. (**a**) Measurement of IFN-γ in blood from day-21 orthotopic HCC mice treated with DC$_{\text{TEX-N1ND}}$ (2x10^6 once per week for 3 weeks) by ELISA on week 3 (n=3), week 5 (n=4; one-way ANOVA post hoc Student-Newman-Keuls test), week 7 (n=5; one-way ANOVA on ranks) and 9 (n=5; two-tailed Mann-Whitney U test) after tumor challenge. (**b**) Measurement of immunosuppressive cytokines including TGF-β and IL-10 in blood from day-21 orthotopic HCC mice treated with DC$_{\text{TEX-N1ND}}$ (2x10^6 once per week for 3 weeks) by ELISA on week 3 (n=3), week 5 (n=4; one-way ANOVA post hoc Student-Newman-Keuls test), week 7 (n=5; one-way ANOVA post hoc Student-Newman-Keuls test) and 9 (n=5; two-tailed t test) after tumor challenge. Data are presented as means ± s.e.m. (*p<0.05; **p<0.001). Source data are provided as a Source Data file.

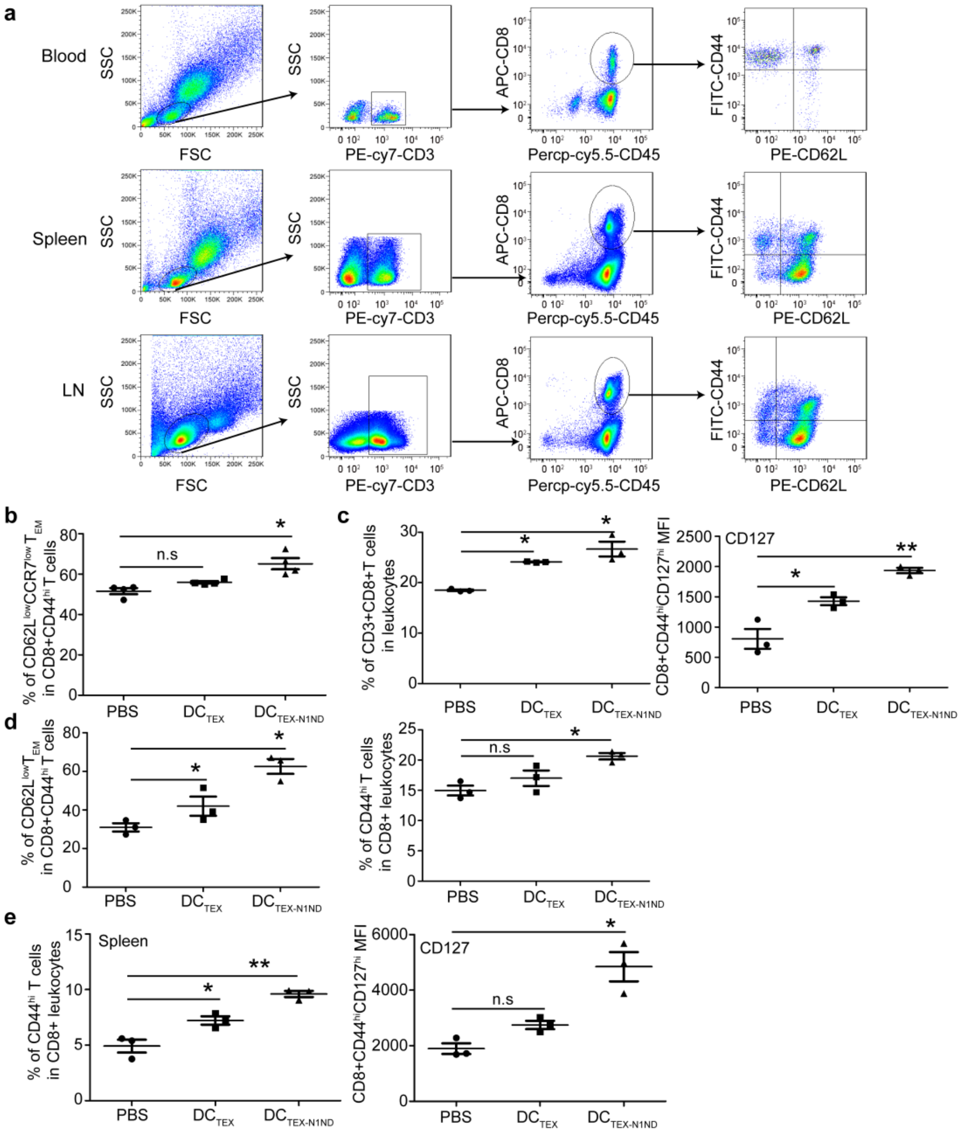

Supplementary Figure 5. Examination of effector and memory T cells in *C57BL/6* mice immunized with DC$_{TEX-N1ND}$ before and after tumor challenge. (**a**) Gating strategy of isolation of memory T cells from different organs presented on Fig. 5a-f, 5i, 6e, Supplementary Fig. 5b-e. (**b**) Flow cytometric analysis of T$_{EM}$ cells in blood from *C57BL/6* mice immunized with DC$_{TEX-N1ND}$, DC$_{TEX}$ (2x10^{6} cells once per week for 3 weeks) or PBS intravenously. Blood was collected 4 weeks after last immunization. CD44^{hi} means CD44^{high} (n=4; one-way ANOVA post hoc Student-Newman-Keuls test). (**c**) Quantitative analysis of effector and long-lived memory T cells and T$_{EM}$ cells (**d**) in blood from *C57BL/6* mice immunized with DC$_{TEX-N1ND}$, DC$_{TEX}$ (2x10^{6} cells once per week for 3 weeks) or PBS 4 weeks after tumor challenge. CD127^{hi} means CD127^{high} (n=3; one-way ANOVA post hoc Student-Newman-Keuls test). MFI: mean fluorescence intensity. (**e**) Quantitative analysis of long-lived memory T cells in spleen from *C57BL/6* mice immunized with DC$_{TEX-N1ND}$, DC$_{TEX}$ (2x10^{6} cells once per week for 3 weeks) or PBS 4 weeks after tumor challenge (n=3; one-way ANOVA post hoc Student-Newman-Keuls test). Data are presented as means ± s.e.m. (*p<0.05; **p<0.001; n.s = not significant). Source data are provided as a Source Data file.

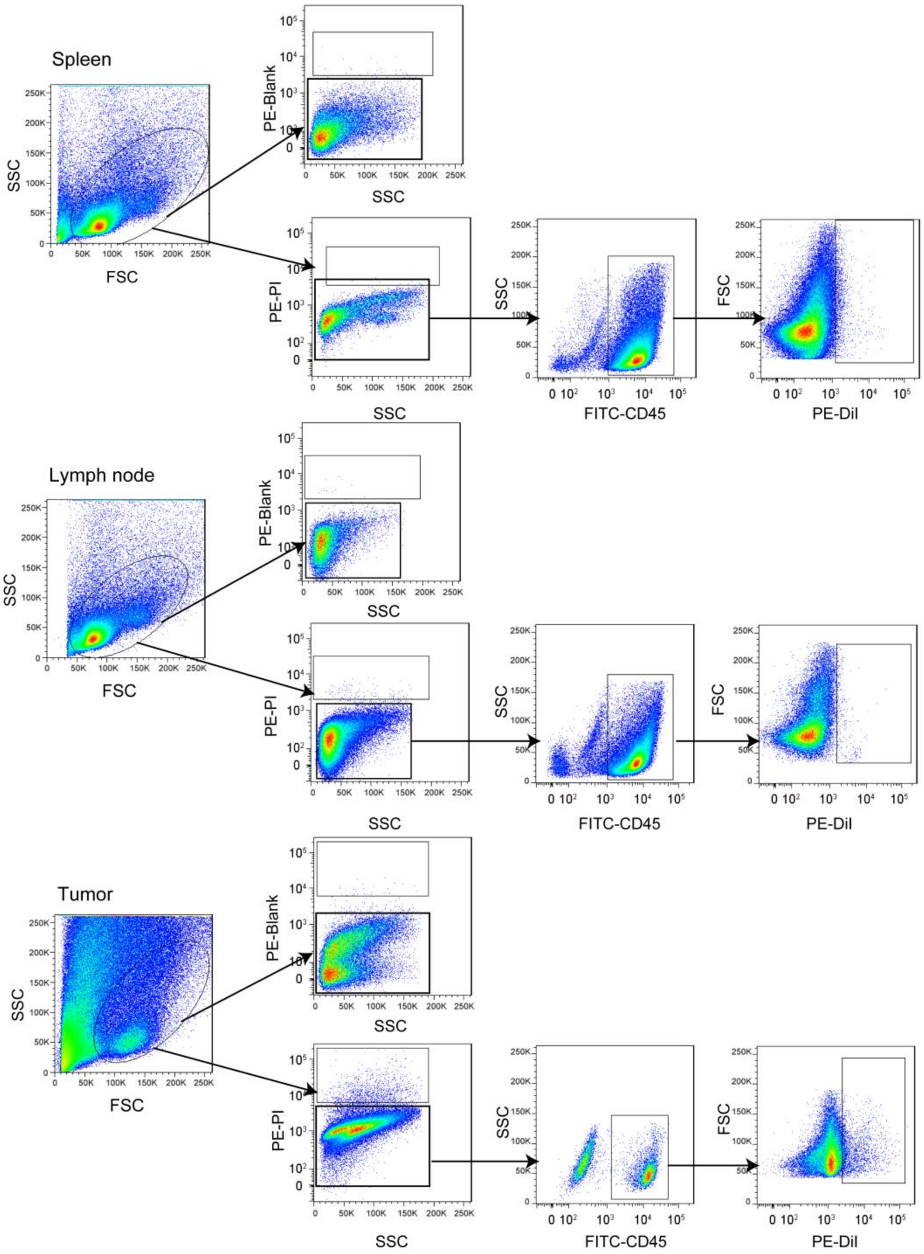

Supplementary Figure 6. Gating strategy for PE-DiI-labelled DC$_{TEX-N1ND}$ from different organs including spleen, lymph nodes and tumor presented on Fig. 6g. DiI-labelled DC$_{TEX-N1ND}$ or DC$_{TEX}$ (5x10^{6}) were administered into orthotopic HCC mice for single intravenous injection and tissues were harvested 48 h post-injection. Source data are provided as a Source Data file.

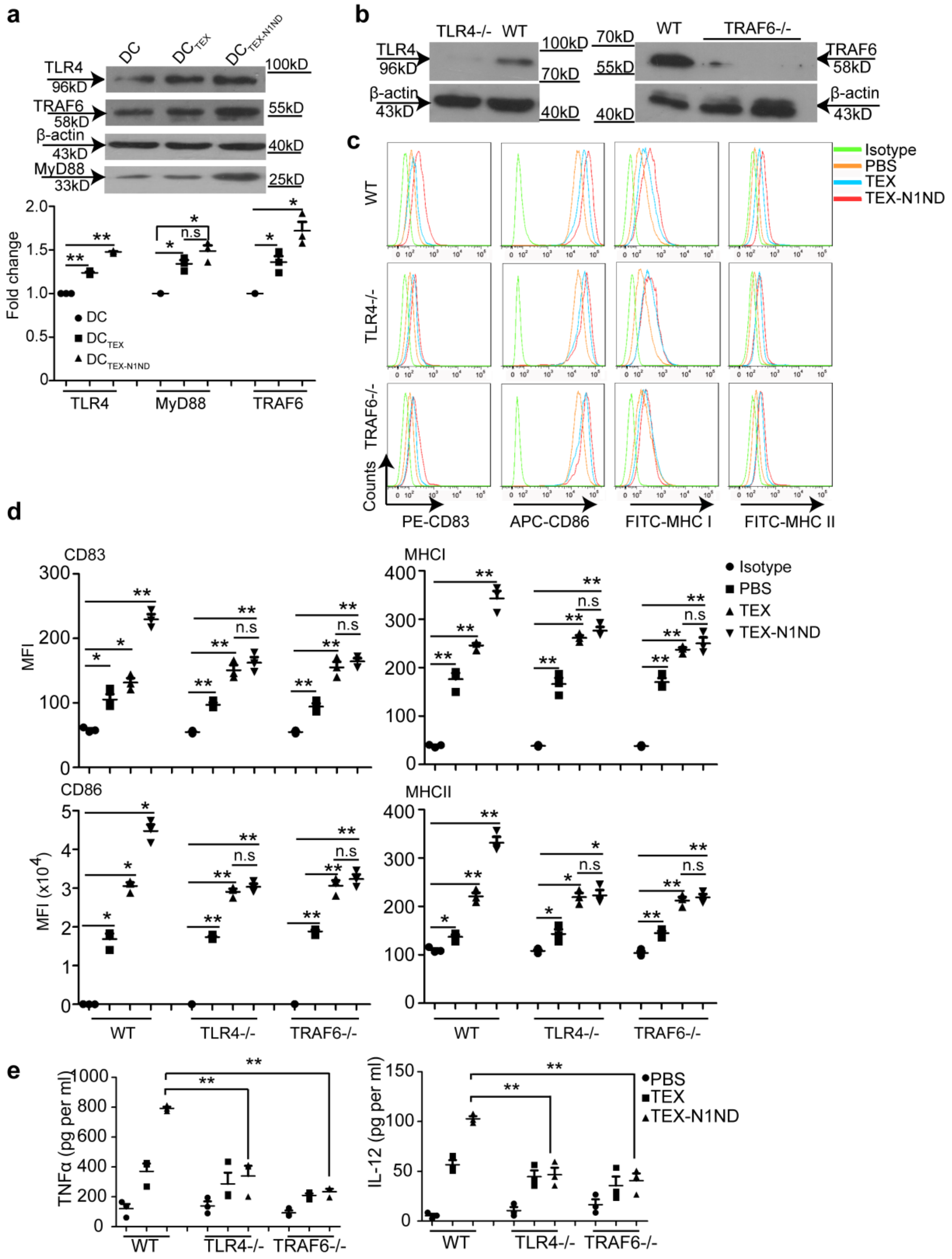

Supplementary Figure 7. Evaluation of TLR4 and TRAF6 in DC$_{TEX\text{-}N1ND}$. (**a**) Western blot analysis for detecting the levels of TLR4, TRAF6 and MyD88 expression in DC, DC$_{TEX}$ or DC$_{TEX\text{-}N1ND}$ (n=3; one-way ANOVA on ranks except for TLR4, in which one-way ANOVA post hoc Student-Newman-Keuls test was used; n represents the number of biological replicates for different cell lines). Total protein (50 μg) was loaded and β-actin was used as a loading control. (**b**) Western blot analysis to determine the expression of TLR4 and TRAF6 on *TLR4*$^{-/-}$ and *TRAF6*$^{-/-}$ DCs. Total protein (50 μg) was loaded and β-actin was used as a loading control. WT refers to wild-type DCs. Flow cytometric (**c**) and quantitative (**d**) analysis of surface markers and co-stimulatory molecules on wild-type (WT), *TLR4*$^{-/-}$ or *TRAF6*$^{-/-}$ DCs

followed by stimulation with TEX or TEX-N1ND (40 μg per ml) for 48 h (n=3; one-way ANOVA post hoc Student-Newman-Keuls test; n represents the number of biological replicates for different cell lines). (**e**) Measurement of TNF-α and IL-2 in the culture medium of wild-type, *TLR4*$^{-/-}$ or *TRAF6*$^{-/-}$ DCs pulsed with TEX or TEX-N1ND for 48 h (n=3; one-way ANOVA post hoc Student-Newman-Keuls test; n represents the number of biological replicates for different cell lines). Data are presented as means ± s.e.m. (*p<0.05; **p<0.001; n.s = not significant). Source data are provided as a Source Data file.

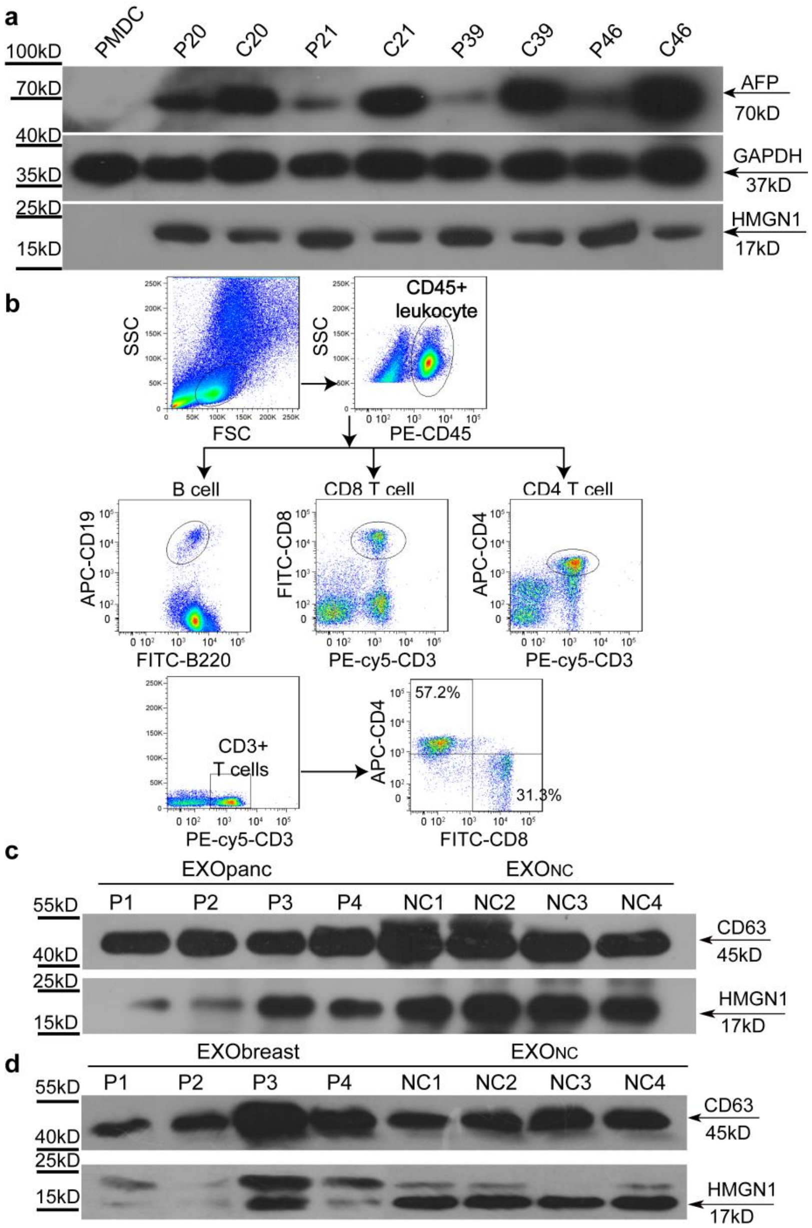

Supplementary Figure 8. Western blot analysis to determine the expression of HMGN1 in HCC patient tissues and exosomes from pancreatic and breast cancer patients' serum. (**a**) Western blot analysis to determine the expression of HMGN1 and AFP in HCC patient tumor and para-tumoral tissues. Total protein (50 μg) was loaded and GAPDH was used as a loading control. PMDC refers to peripheral monocyte-derived DCs and was used as a negative control. P and C represent Para-tumoral and Cancer tissues, respectively. This experiment was repeated once (two in total). (**b**) Gating strategy for human PBMC-derived lymphocytes. Western blot analysis to determine the expression of HMGN1 in exosomes derived from pancreatic (**c**) and breast (**d**) cancer patients' serum. Total protein (20 μg) was loaded and CD63 was used as an exosomal marker. EXO_{panc}, EXO_{breast} and EXO_{NC} refer to exosomes derived from pancreatic, breast cancer patients' and normal volunteers' serum. P and NC represent patients and normal controls, respectively. Source data are provided as a Source Data file.

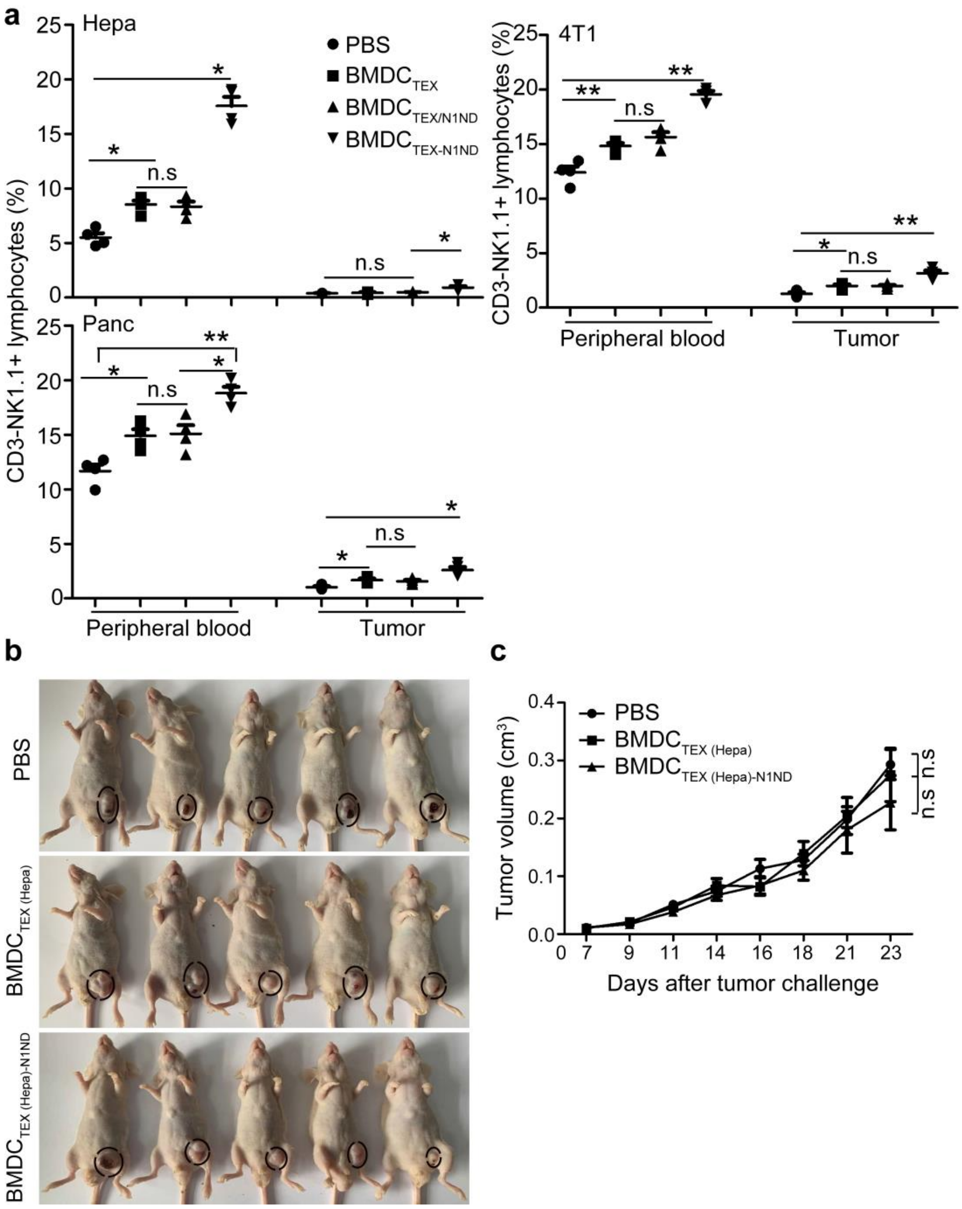

Supplementary Figure 9. Examination of immune responses in different subcutaneous tumor mouse models. (**a**) Flow cytometric analysis of NK cells in blood and tumor tissues from different subcutaneous HCC (one-way ANOVA on ranks), pancreatic and breast (one-way ANOVA post hoc Student-Newman-Keuls test) tumor mouse models treated with BMDC$_{TEX-N1ND}$, BMDC$_{TEX\ /\ N1ND}$ or BMDC$_{TEX}$ (2x10^6 cells once per week for 3 weeks) 42 days after tumor challenge (n=4). Representative tumor images (**b**) or measurement of tumor volume (**c**) of day-7 established subcutaneous HCC nude mice treated with DC$_{TEX(Hepa)-N1ND}$ or DC$_{TEX(Hepa)}$ (2x10^6 cells once per week for 3 weeks) at different time-points (n=5; one-way ANOVA post hoc Student-Newman-Keuls test). Data are presented as means ± s.e.m. (*p<0.05; **p<0.001; n.s = not significant). Source data are provided as a Source Data file.

Supplementary Figure 10 Uncropped Original Scans

Figure 1a

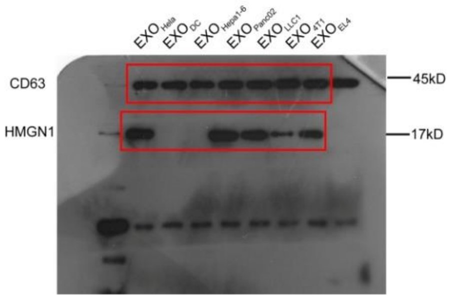

Figure 7a

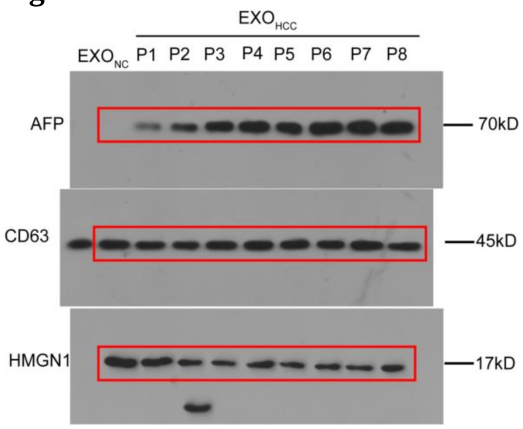

Supplementary Figure 1a

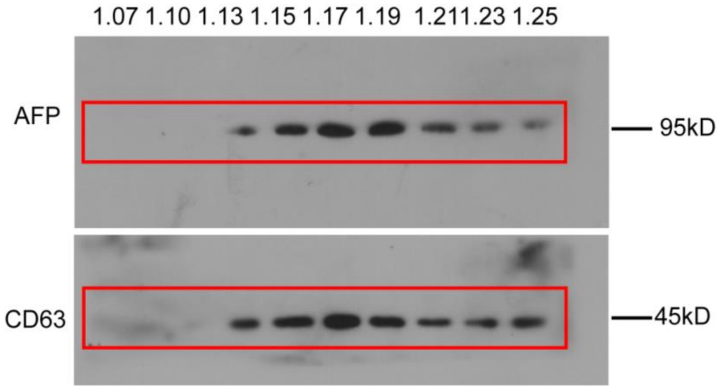

Supplementary Figure 1b

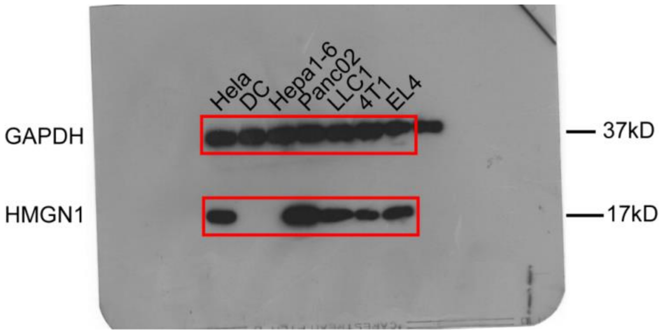

Supplementary Figure 7a

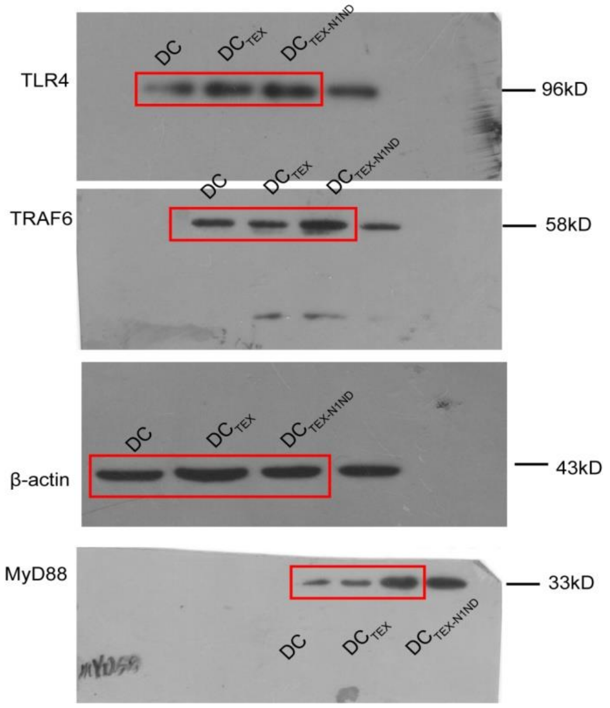

Supplementary Figure 7b

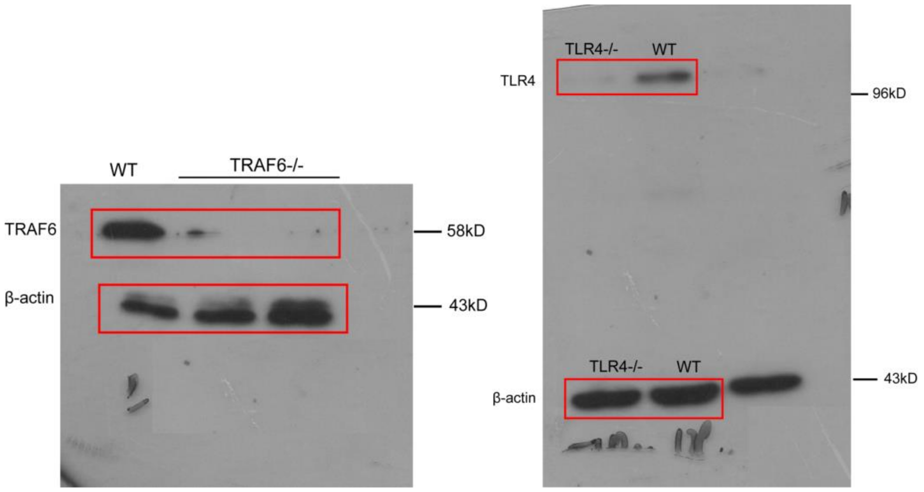

Supplementary Figure 8a

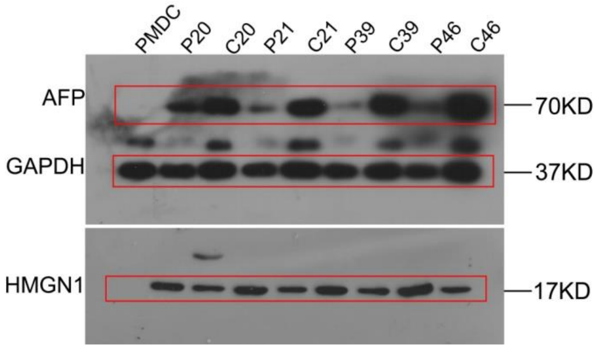

Supplementary Figure 8c

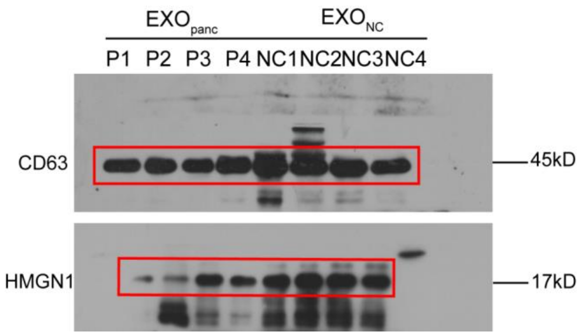

Supplementary Figure 8d

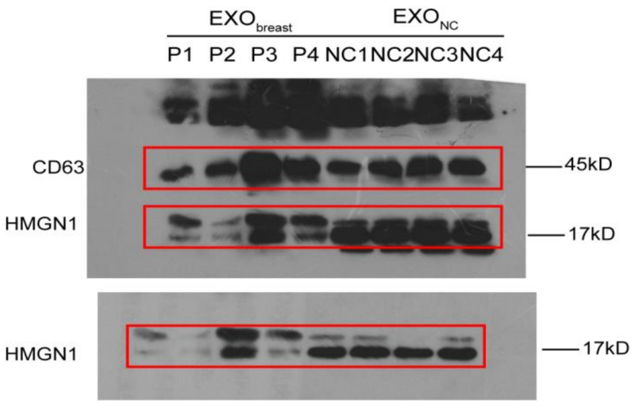

Supplement: Supplementary file 1 — Supplementary Information [file 41467_2020_15569_MOESM1_ESM.pdf]
