# Supplementary material for: Alarmin-painted exosomes elicit persistent antitumor immunity in large established tumors in mice
Source: Nat Commun. 2020 Apr 14;11:1790. doi: 10.1038/s41467-020-15569-2 (PMC7156382; doi:10.1038/s41467-020-15569-2)
Supplement: Supplementary file 2 — Reporting Summary [file 41467_2020_15569_MOESM2_ESM.pdf]

nature research

Corresponding author(s): HaiFang Yin

Last updated by author(s): 26/02/20

Reporting Summary

Nature Research wishes to improve the reproducibility of the work that we publish. This form provides structure for consistency and transparency in reporting. For further information on Nature Research policies, see Authors & Referees and the Editorial Policy Checklist .

Statistics

For all statistical analyses, confirm that the following items are present in the figure legend, table legend, main text, or Methods section.

n/a	Confirmed	
[ ]	[x]	The exact sample size (n) for each experimental group/condition, given as a discrete number and unit of measurement
[ ]	[x]	A statement on whether measurements were taken from distinct samples or whether the same sample was measured repeatedly
[ ]	[x]	The statistical test(s) used AND whether they are one- or two-sided<br>*Only common tests should be described solely by name; describe more complex techniques in the Methods section.*
[x]	[ ]	A description of all covariates tested
[x]	[ ]	A description of any assumptions or corrections, such as tests of normality and adjustment for multiple comparisons
[ ]	[x]	A full description of the statistical parameters including central tendency (e.g. means) or other basic estimates (e.g. regression coefficient) AND variation (e.g. standard deviation) or associated estimates of uncertainty (e.g. confidence intervals)
[ ]	[x]	For null hypothesis testing, the test statistic (e.g. F, t, r) with confidence intervals, effect sizes, degrees of freedom and P value noted<br>*Give P values as exact values whenever suitable.*
[x]	[ ]	For Bayesian analysis, information on the choice of priors and Markov chain Monte Carlo settings
[x]	[ ]	For hierarchical and complex designs, identification of the appropriate level for tests and full reporting of outcomes
[x]	[ ]	Estimates of effect sizes (e.g. Cohen's d, Pearson's r), indicating how they were calculated

Our web collection on statistics for biologists contains articles on many of the points above.

Software and code

Policy information about availability of computer code

Data collection	Flow cytometry data were collected using BD LSRFortessa and analyzed with FlowJo version 7.6 (Tree Star, US).
Data analysis	Statistical differences between treatment and control groups were evaluated by SigmaStat v3.5 (Systat Software, London, UK). Sample size was determined by G*Power 3.1.7.

For manuscripts utilizing custom algorithms or software that are central to the research but not yet described in published literature, software must be made available to editors/reviewers. We strongly encourage code deposition in a community repository (e.g. GitHub). See the Nature Research guidelines for submitting code & software for further information.

Data

Policy information about availability of data

All manuscripts must include a data availability statement. This statement should provide the following information, where applicable:

- Accession codes, unique identifiers, or web links for publicly available datasets
- A list of figures that have associated raw data
- A description of any restrictions on data availability

The source data underlying Figs 1a, 1e-h, 2a-g, 3b, 3d-e, 4a-b, 4d-f, 5a-j, 6a-g, 7a-d and Supplementary Figs 1a-b, 2d-f, 3a, 3d-e, 4a-b, 5b-e, 7a-b, 7d-e, 8a, 8c-d, 9a and 9c are provided as a Source Data file. All the other data supporting the findings of this study are available within the article and its supplementary information files and from the corresponding author upon reasonable request.

Field-specific reporting

Please select the one below that is the best fit for your research. If you are not sure, read the appropriate sections before making your selection.

Life sciences study design

All studies must disclose on these points even when the disclosure is negative.

Sample size	The sample sizes were determined based on pilot studies and previous publications (Rao, et al. Hepatology (2016) 64(2):456-472); Lu et al. Journal of Hepatology (2017) 67:739–748). Most experiments were performed multiple times. If results were reproducible, the conclusions were consider valid.
Data exclusions	no data were excluded from the study.
Replication	all attempts at replication were successful. How many times each experiment was performed and which statistical analysis used is indicated in the figure legends.
Randomization	all samples were randomly allocated into experimental groups.
Blinding	the investigators were not blinded to the group allocation during data collection and / or data analysis because all samples were analyzed in the same way.

Reporting for specific materials, systems and methods

We require information from authors about some types of materials, experimental systems and methods used in many studies. Here, indicate whether each material, system or method listed is relevant to your study. If you are not sure if a list item applies to your research, read the appropriate section before selecting a response.

Materials & experimental systems

n/a	Involved in the study	
☐	☒	Antibodies
☐	☒	Eukaryotic cell lines
☒	☐	Palaeontology
☐	☒	Animals and other organisms
☐	☒	Human research participants
☒	☐	Clinical data

Methods

n/a	Involved in the study	
☒	☐	ChIP-seq
☐	☒	Flow cytometry
☒	☐	MRI-based neuroimaging

Antibodies

Antibodies used

Antibodies are listed below as: target label (company, catalog number, dilution)
FITC- anti-mouse IgG2b Isotype (Biolegend,402207)1:500
PE- anti-mouse IgG2b Isotype (Biolegend,402203)1:500
PE- -cy7anti-mouse IgG2b Isotype (eBioscience,25-4031-81)1:500
APC- anti-mouse IgG2b Isotype (Biolegend,402205)1:500
FITC-anti-mouse MHCI(eBioscience,11-5958-80)1:250
FITC-anti-mouse MHCII (Biolegend,116405)1:250
FITC -anti-mouse CD80 (abcam,ab24860)1:250
PE- anti-mouse CD83 (Biolegend,121507)1:250
APC-anti-mouse CD86 (eBioscience,17-0862-82)1:250
PE-cy5.5 anti-mouse CD11c (eBioscience,35-0114-82)1:250
FITC-anti-mouse CD45(Biolegend,103107)1:500
percp-cy5.5-anti-mouse CD45(Biolegend,103131)1:500
FITC-anti-mouse CD3e(eBioscience,11-0031-82)1:500
PE-anti-mouse CD3e(eBioscience, 12-0031-82)1:500
PE-cy7-anti-mouse CD3e(eBioscience, 25-0032-80)1:500
PE-anti-mouse CD4 (eBioscience,12-0041-83)1:500
APC-anti-mouse CD8a (Biolegend,100712)1:500
PE-anti-mouse CD11c (Biolegend,117308)1:250
BV421-anti-mouse CD11b(Biolegend,101236)1:500
FITC-anti-mouse B220/CD45R(eBioscience,11-0452-81)1:500
PE-cy7anti-mouse CD19(Biolegend,115519)1:500
APC-anti-mouse-NK1.1(eBioscience,17-5941-81)1:500
PE-cy7-anti-mouse CD25(eBioscience,25-0251-81)1:500
PE-anti-mouse CD62L(eBioscience,12-0621-81)1:500
FITC-anti-mouse CD44(eBioscience,11-0441-81)1:500
PE-cy7anti-mouse CD127(eBioscience,25-1273-80)1:500

eFluro450-anti-mouse CCR7(eBioscience,48-1971-80)1:250
PE- anti- human CD45(Biolegend,304007)1:500
PE-cy5-anti- human CD3e(Biolegend,300309)1:500
APC-anti- human CD4(Biolegend,357407)1:500
FITC-anti-human B220/CD45R(eBioscience,11-0452-81)1:500
FITC-anti- human CD8a (Biolegend,301006)1:500
FITC-anti- human -CD56 (Biolegend,318303)1:500
APC-anti- human CD19(Biolegend,302211)1:500
PE-cy7anti-mouse-CD63(eBioscience,25-0631-80)1:500
PE-cy7 rat IgG2a isotype((eBioscience,-25-4031-82)1:500

western blotting antibody:
β-actin (Cell Signaling Technology,#4970s)1:2000
HMGN1 ((ProteinTech; 11695-1-AP)1:1000
CD63 (Santa Cruz,sc-5275)1:200
Alix (Cell Signaling Technology,, #2171)1:200
GAPDH (Multisciences, 70-ab36233-100)1:1000
TLR4(Proteintech,19811-1-AP)1:1000
myD88(Cell signaling Technology,#4283)1:1000
TRAF6(abcam,ab33915)1:5000
AFP(abcam,ab46799)1:2000

IHC antibody:
CD3(Novus,NBP1-96545)1:2000
Foxp3(abcam,ab54501)1:2000

HRP labeled Secondary Antibody,:
peroxidase-conjugated goat anti-mouse IgG (Sigma,US,31430),1:5000
peroxidase-conjugated goat anti-rabbit IgG (Sigma, US,31460),1:5000
peroxidase-conjugated rabbit anti-goat IgG (Sigma, US,31402),1:5000

Validation

Antibody validation was referred to the manufacturers and was supported by multiple publications.

Eukaryotic cell lines

Policy information about cell lines

Cell line source(s)

Murine dendritic cell line DC2.4 was provided by Dr De Yang, Center for Cancer Research, NIH, US. Murine HCC cell line Hepa1-6 was purchased from Boster Biological Technology Ltd (Wuhan, China). Murine pancreatic cancer cell line (Panc02, H-2b), Lewis lung cancer cell line (LLC1, H-2b), breast cancer cell line (4T1, H-2d), cervical cancer cell (Hela) and lymphoma cell (EL4) lines were kept in house and originally purchased from ATCC biobank. Human HCC cell lines including HepG2, Hep3B, Panc1 and MCF7 were purchased from ATCC biobank; MHCC -97H was purchased from Shanghai Institute for Biological Sciences, Chinese Academy of Sciences and LM3 was purchased from BeNa Culture Collection, Beijing, China.

Authentication

none of the cell lines used were authenticated

Mycoplasma contamination

all cell lines were tested negative for mycoplasma contamination

Commonly misidentified lines
(See ICLAC register)

none

Animals and other organisms

Policy information about studies involving animals; ARRIVE guidelines recommended for reporting animal research

Laboratory animals

6-8 week old C57BL/6J wild-type (H-2b) , BALB/C (H-2d) and thymus-deficient BALB/C nude mice were purchased from Beijing Vital River Laboratory Animal Technology Co., Ltd. (Beijing, China). Both male and female mice were used for the study.

Wild animals

the study did not involve any wild animal.

Field-collected samples

the study did not involve samples collected from the field.

Ethics oversight

All the animal experiments were carried out in the animal unit, Tianjin Medical University (Tianjin, China) according to procedures authorized and specifically approved by the institutional ethical committee (Permit Number: SYXK 2009-0001).

Note that full information on the approval of the study protocol must also be provided in the manuscript.

Human research participants

Policy information about studies involving human research participants

Population characteristics	Blood and serum samples from healthy volunteers were purchased from Tianjin Blood center (Tianjin, China), which is a commercial source. Serum samples from cancer patients were purchased from biobank of Tianjin Medical University Cancer Institute and Hospital (Tianjin, China, with the approval of the hospital ethic committee and Tianjin science and technology commission (Permit number (2016)621).
Recruitment	*Describe how participants were recruited. Outline any potential self-selection bias or other biases that may be present and how these are likely to impact results.*
Ethics oversight	approved by the hospital ethic committee and Tianjin science and technology commission (Permit number (2016)621).

Note that full information on the approval of the study protocol must also be provided in the manuscript.

Flow Cytometry

Plots

Confirm that:

- [x] The axis labels state the marker and fluorochrome used (e.g. CD4-FITC).
- [x] The axis scales are clearly visible. Include numbers along axes only for bottom left plot of group (a 'group' is an analysis of identical markers).
- [x] All plots are contour plots with outliers or pseudocolor plots.
- [x] A numerical value for number of cells or percentage (with statistics) is provided.

Methodology

Sample preparation	Samples were prepared as described in the methods section. Animals were culled according to procedures authorized and specifically approved by the institutional ethical committee (permit SYXK 2009-0001).The single cell suspensions were prepared from spleen and LN's by mashing tissues through a 70 μm cell strainer. Human peripheral blood was obtained from healthy volunteers (provided by Tianjin Blood Center, Tianjin, China). Single cell suspensions from liver or tumor tissues were minced into small pieces with surgical scissors, were digested in collagenase type IV suspension (0.05 mg/ml, Worthington Biochem. Corp., NJ, US); filtered through the 70 μm cell strainer, then the mixture was re-suspended with 40% percoll (Pharmacia, Sweden) to remove the supernatant and with the ACK lysis buffer to remove red blood cells. Live/Dead stains were used to exclude dead cells.Peripheral blood mononuclear cells were isolated with human Lymphoprep solution (Axis-shield PoC AS, Oslo,Norway) per the manufacturer's instruction.Single cell suspensions collected for further analysis by flow cytometry.
Instrument	All data were collected on BD LSRFortessa instrument (BD Biosciences)
Software	Data analysis was performed using FlowJo version 7.6 (Tree Star, USA)
Cell population abundance	For all cell populations analysed, abundances are indicated in the figure plots.
Gating strategy	All gate strategies captured cells by FSC vs SSC area, single cells by FSC height versus area.<br>The PI- negative cells were gated as live cells for further analysis. Gating strategies beyond this differed by experiment.

- [x] Tick this box to confirm that a figure exemplifying the gating strategy is provided in the Supplementary Information.
